# Serine Grafted Silica Coated Nanoscale Zero‐Valent Iron with Enhanced Fenton‐Like Degradation of Mixed Organic Solvents of Tributyl Phosphate and n‐dodecane

**DOI:** 10.1002/advs.202509319

**Published:** 2025-08-11

**Authors:** Peijie Sun, Haifeng Xu, Lejin Xu

**Affiliations:** ^1^ Department of Nuclear Engineering and Technology School of Energy and Power Engineering Huazhong University of Science and Technology Wuhan 430074 P. R. China

**Keywords:** fenton‐like catalyst, nanoscale zero‐valent iron, n‐dodecane, serine, silica, tributyl phosphate

## Abstract

As global nuclear energy scales up, the annual output of low‐to‐medium‐level radioactive tributyl phosphate (TBP) and n‐dodecane organic solvents from spent fuel processing grows. To address this problem, the serine grafted silica coated nanoscale zero‐valent iron (Ser‐SiO_2_@nZVI) is synthesized and used as a Fenton‐like catalyst. Ser‐SiO_2_@nZVI is analyzed through various characterization techniques. The catalytic oxidation performance of Ser‐SiO_2_@nZVI in the Fenton‐like system is evaluated. The effects of catalyst type, temperature, catalyst dosage, H_2_O_2_ concentration and H_2_SO_4_ concentration on the degradation of organic solvents are optimized. Nuclear isotope simulation experiments proved that the presence of nuclides do not affect the degradation efficiency, and more than 90% of the nuclides remained in the reaction vessel. Electron spin resonance (ESR) results indicated that the Ser‐SiO_2_@nZVI/H_2_O_2_ system produced more hydroxyl radicals than the nZVI/H_2_O_2_ system. Density functional theory (DFT) calculations further suggested that the catalytic performance of Ser‐SiO_2_@nZVI is higher, and the adsorption energy between Ser‐SiO_2_@nZVI and TBP/n‐dodecane is greater, making it more likely to adsorb organic solvents on the catalyst surface. This research provides a theoretical basis for the modification of iron‐based materials and their application in the treatment of low‐to‐medium radioactive organic solvents.

## Graphical abstract

Serine grafted silica coated nanoscale zero‐valent iron is synthesized. Through the SiO_2_ coating and serine grafting, the aggregation and oxidation of nZVI are reduced, and the slow release of Fe^2+^ ensured the catalytic cycle. The adsorption capacity for TBP and n‐dodecane is higher, because of the synergistic effect of surface functional groups. The HOMO‐LUMO gap is smaller and more •OH are generated, improving the catalytic activity.

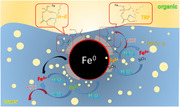

## 1 Introduction

Nuclear energy is an energy source with great potential as a substitute for dwindling fossil fuels, and the reprocessing of spent nuclear fuel is a major challenge in the development of nuclear energy.^[^ ^1^, ^2^ ^]^ In the reprocessing of spent nuclear fuel, that is, the extraction of uranium and plutonium from spent nuclear fuel for further use as nuclear fuel,^[^ ^3^, ^4^ ^]^ tributyl phosphate (TBP) has been widely used as an extractant in plutonium uranium recovery by extraction (PUREX) process.^[^ ^5^ ^]^ In the PUREX process, TBP can extract uranium and plutonium in the +6 and +4 oxidized states in the form of UO_2_(NO_3_)_2_‐2TBP and Pu(NO_3_)_4_‐2TBP, respectively, from the solution of spent nuclear fuel dissolved in nitric acid.^[^ ^6^, ^7^ ^]^ At the same time, diluents such as n‐dodecane and kerosene are added to TBP, partly because the density of pure TBP is close to that of aqueous solution, and partly to depress the maximum metal load of TBP.^[^ ^8^, ^9^ ^]^ The most commonly used diluent is n‐dodecane, which accounts for 70% by volume.^[^ ^10^ ^]^ With the increase of using time, the extraction capacity of TBP decreases gradually.^[^ ^11^ ^]^ After the extraction capability is exhausted, the mixture is removed from the operation and classified as radioactive waste.^[^ ^12^, ^13^ ^]^ TBP is also a stable compound that will exist in soil and water for a long time, so it is necessary to properly treat TBP before safe disposal.^[^ ^14^, ^15^ ^]^

Several methods have been used to handle TBP, such as biological treatment,^[^ ^16^ ^]^ incineration,^[^ ^14^, ^17^ ^]^ vitrification,^[^ ^18^ ^]^ cement solidification^[^ ^19^, ^20^ ^]^ and advanced oxidation processes. Biological treatment is the use of mixed culture or single plant culture microorganisms to treat TBP. There are no environmental problems associated with the formation of secondary byproducts in the biological treatment of TBP, but biological methods require longer processing times.^[^ ^21^, ^22^, ^23^ ^]^ Incineration is a common TBP treatment method, but the incineration of TBP will produce corrosive product P_2_O_5_ as a secondary pollutant, leading to difficult disposal.^[^ ^14^, ^24^ ^]^ Vitrification also has the disadvantages of complex operation and high temperature.^[^ ^12^, ^25^ ^]^ Cement solidification has the advantages of simple process, low cost, normal temperature and pressure operation, but TBP is not miscible with water, resulting in difficult to be solidified by cement.^[^ ^20^ ^]^ Advanced oxidation processes (AOPs) are highly attractive and efficient for removing refractory organic matter,^[^ ^26^, ^27^ ^]^ which can produce highly reactive and non‐selective hydroxyl radicals to decompose the target compounds into non‐toxic intermediates.^[^ ^27^ ^]^ Advanced oxidation methods such as ultraviolet/H_2_O_2_ treatment^[^ ^27^, ^28^ ^]^ and underwater plasma^[^ ^14^ ^]^ have been used to degrade TBP.

Fenton oxidation is an advanced oxidation process with low reaction temperature, atmospheric pressure, high efficiency, low cost and no dangerous gas emission.^[^ ^29^, ^30^, ^31^ ^]^ The Fenton reaction produces hydroxyl radicals by the reaction of hydrogen peroxide with ferrous ions.^[^ ^32^, ^33^, ^34^ ^]^ Homogeneous Fenton reaction can only be carried out under low pH values, and a large amount of reagent solution will be introduced and a large amount of iron mud will be produced.^[^ ^35^ ^]^ Heterogeneous Fenton process can avoid some disadvantages of homogeneous Fenton.^[^ ^36^, ^37^ ^]^ Nano zero‐valent iron (nZVI) has been proved to be an excellent catalyst for heterogeneous Fenton reaction with large specific surface area, high catalytic activity and certain adsorption properties.^[^ ^38^, ^39^ ^]^ The nZVI particles tend to aggregate into larger size particles due to van der Waals forces and magnetic forces, resulting in reduced mobility of nZVI, which is also easily oxidized in aerobic environments, resulting in significant reduced reactivity.^[^ ^40^, ^41^, ^42^, ^43^ ^]^ Silica is an excellent material for the protection of nZVI particles due to its high chemical stability and low toxicity.^[^ ^44^, ^45^ ^]^ Silica coated nZVI has the characteristics of high reduction efficiency, no secondary pollution and good biocompatibility, and its ability to degrade some pollutants is much higher than that of bare nZVI.^[^ ^46^, ^47^, ^48^ ^]^ In addition, there are abundant hydroxyl groups on the surface of silica, which provides convenience for subsequent surface modification and grafting of functional groups.^[^ ^45^, ^49^ ^]^ Amino acids are excellent surface modification materials with at least one alkaline amino and one highly active and hydrophilic acidic carboxyl group, which can provide the required characteristics for the surface modification of materials.^[^ ^50^, ^51^, ^52^ ^]^ Our research group^[^ ^50^ ^]^ have calculated, synthesized and characterized a series of novel amino acid‐modified Fe° catalysts, which have been applied to Fenton‐like degradation of TBP and n‐dodecane. The results showed that serine modified nano zero‐valent iron had the most stable structure, the highest activity and the best degradation effect.

First principles calculations, particularly those using density functional theory (DFT) methods, have become powerful techniques for investigating molecular structures and understanding the properties of organic/inorganic interfaces.^[^ ^53^ ^]^ Consequently, computational simulations of DFT have been used to study the adsorption of organic molecules or polyelectrolytes at mineral interfaces at the molecular and atomic levels.^[^ ^53^, ^54^ ^]^ Fang et al.^[^ ^55^ ^]^ calculated the adsorption energy of nZVI for perfluorooctanoic acid (PFOA) and perfluorooctane sulfonate (PFOS) using DFT, and their results indicated that the adsorption energy of PFOS by Fe (0) was higher than that of PFOA, which aligned with the experimental characterization results. Li et al.^[^ ^56^ ^]^ investigated the adsorption mechanism of nitrogen‐containing benzene derivatives on oxygen‐functional group‐modified activated carbon using both experimental and DFT methods. The adsorption energy of alkaline nitrogen‐containing compounds, such as aniline and quinolone, ranged from 7.32 to 7.38 kJ mol^−1^, which was higher than that of neutral nitrogen‐containing compounds like indole, carbazole, and 3‐ethylcarbazole, with adsorption energies ranging from ≈4.28 to 4.83 kJ mol^−1^. Yang et al.^[^ ^35^ ^]^ employed DFT calculations to determine the adsorption energy of TBP and n‐dodecane by nZVI. Therefore, calculating the adsorption energy of catalytic materials and organic solvents using DFT is helpful to analyze the adsorption and degradation mechanisms of organic solvents.

In order to efficiently and safely handle the radioactive organic solvents generated during the post‐processing of spent fuel and to explore the reaction mechanism during the degradation process, nZVI was modified to obtain a more efficient Fenton‐like catalyst for the treatment of organic solvents. The treatment process was safely investigated through simulated nuclear isotope experiments. The objective of this study was to develop a novel serine grafted silica coated nanoscale zero‐valent iron catalyst (Ser‐SiO_2_@nZVI) by the serine grafting and Stöber method on the surface of nZVI, which was used to degrade simulated radioactive organic solvents such as TBP and n‐dodecane. The optimal reaction conditions for the degradation experiment were explored. Elements such as Nd^3+^, Ce^4+^, Sr, and U were used to simulate radioactive nuclides to study the safety of the treatment process. Finally, the degradation mechanism of the reaction process was studied through characterization and DFT simulation.

## 2 Results and Discussion

### 2.1 Characterization and Simulation of Catalysts

**Figure 1:**
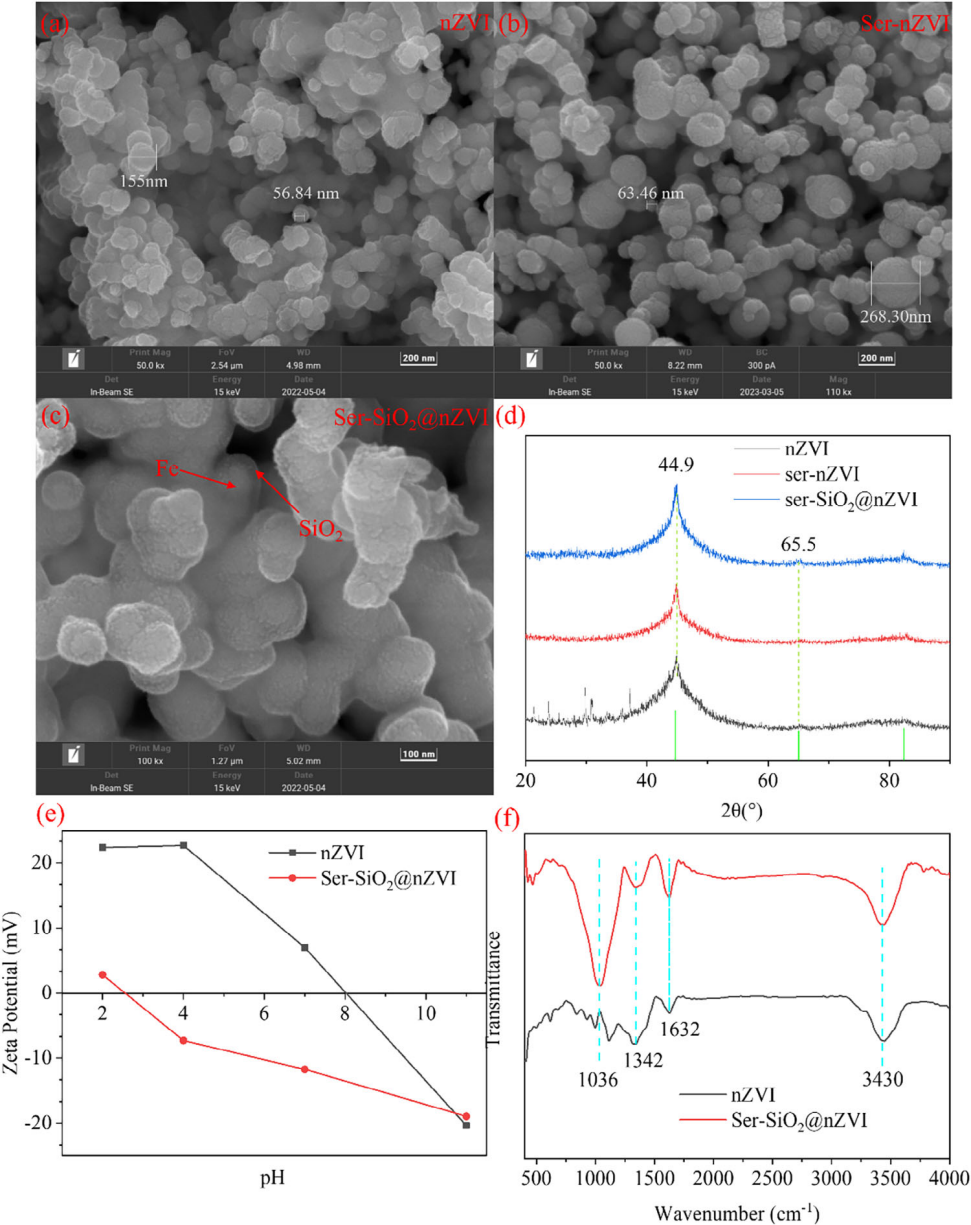
SEM images (200 nm) of a) nZVI and b) Ser‐nZVI; SEM image (100 nm) of c) Ser‐SiO_2_@nZVI; d) XRD patterns of nZVI, Ser‐nZVI and Ser‐SiO_2_@nZVI; (d) XRD patterns of Ser‐SiO_2_@nZVI before and after adsorption or reaction; e) Zeta potentials of nZVI and Ser‐SiO_2_@nZVI at different pH values; f) FTIR spectra of nZVI and Ser‐SiO_2_@nZVI.

The properties of zero‐valent iron modified by serine grafting and silica coating were studied by characterization analysis and DFT simulation. As shown in **Figure** **1**a,b, both nZVI and Ser‐nZVI exhibit relatively regular spherical shapes. The particle size distribution of nZVI is between 50 and 160 nm, while the particle size of Ser‐nZVI is slightly larger, ranging from 60 to 270 nm. Compared with previous studies, the particle size of the nanomaterials obtained in this research is larger, but its impact on the specific surface area and catalytic performance is relatively small. This is because these materials have almost no pores, and the adsorption of the target substance is based on the type of surface functional groups rather than the number of adsorption sites.^[^ ^35^ ^]^ Ser‐SiO_2_@nZVI in Figure 1c shows a relatively obvious core/shell structure, with a zero valent iron core inside with a coated silica outside.^[^ ^49^ ^]^ As seen in **Table** **1**, compared with nZVI (Figure S1, Supporting Information), the increase of N element in the EDX spectrum of Ser‐nZVI (Figure S2, Supporting Information) indicates that serine has been successfully grafted onto Fe^0^, while the presence of Si element in Ser‐SiO_2_@nZVI (Figure S3, Supporting Information) and the increase of N element indicate that silica has been successfully coated on the surface of Fe^0^, and serine has also been successfully grafted.

**Table 1:** Energy dispersive spectroscopy (EDS) patterns of chemical composition of nZVI, Ser‐nZVI, Ser‐SiO_2_@nZVI, Ser‐SiO_2_@nZVI before, after adsorption and after reaction.

Elements	nZVI	Ser‐nZVI	Ser‐SiO_2_@nZVI		
			Initial	Adsorption	Reaction
O (wt.%)	4.01	5.76	15.90	15.05	43.86
Fe (wt.%)	95.99	93.58	75.68	76.49	39.16
N (wt.%)	−	0.66	0.34	0.44	0.13
Si (wt.%)	−	−	8.09	7.60	6.06
P (wt.%)	−	−	−	0.41	10.79

As shown in Figure S6 (Supporting Information), the specific surface areas and characteristic porosities of nZVI, Ser‐nZVI and Ser‐SiO_2_@nZVI can be obtained from nitrogen adsorption/desorption isotherms and BJH pore size distribution curves. The nitrogen adsorption/desorption isotherms of nZVI, Ser‐nZVI and Ser‐SiO_2_@nZVI all show a type IV hysteresis loop of H3, indicating that the catalysts have typical mesoporous structures.^[^ ^35^, ^57^ ^]^ As can be seen from Table S1 (Supporting Information), the specific surface areas and pore volumes of the catalysts are relatively small, indicating that the adsorption of organic solvents by the catalysts is based on the types of surface functional groups rather than the number of adsorption sites. As measured and presented in Figure S7 (Supporting Information), the water contact angles of nZVI, Ser‐nZVI and Ser‐SiO_2_@nZVI are 28.9°, 29.6°, and 32.9°, respectively. After the serine was grafted, the surface hydrophobicity of the catalyst remained basically unchanged. This was because the number of serine grafts was relatively small, and serine itself is a hydrophilic amino acid. However, after the silica coating, the hydrophobicity of the catalyst increased, indicating that the silica successfully covered the surface of Fe^0^, resulting in an enhanced hydrophobicity.^[^ ^58^ ^]^ With stronger hydrophobicity, it can increase the distance between H_2_O and catalyst surface, thus inhibiting surface passivation, improving catalytic activity and increasing use time.^[^ ^59^ ^]^

The XRD pattern (Figure 1d) shows the characteristic diffraction patterns of nZVI, Ser‐nZVI and Ser‐SiO_2_@nZVI, which contain a peak at 44.67° and can be attributed to the (110) plane (d = 2.0268). The weak peak at 65.17° corresponds to the (200) plane of the body‐centered cubic a‐Fe (JCPDS No. 06‐0696) diffraction pattern.^[^ ^60^, ^61^ ^]^ It should be noted that there are some impurity peaks at 2θ = 30–40° in the XRD spectrum of nZVI, confirming the formation of iron oxide.^[^ ^62^, ^63^ ^]^ However, similar peaks were not observed in Ser‐nZVI and Ser‐SiO_2_@nZVI, indicating that grafting serine onto the surface or coating with silica can reduce the oxidation of Fe^0^.

Figure 1e presents the zeta potentials of nZVI and Ser‐SiO_2_@nZVI at different pH values. The surface charge neutral pH of the catalyst is the isoelectric point (IEP). The IEP of nZVI was close to pH 8.0, which is close to the IEP value (8.3) of nanometer zero‐valent iron reported in the literature.^[^ ^61^, ^64^ ^]^ The IEP of Ser‐SiO_2_@nZVI was about pH 2, which is close to the IEP of silica, indicating that there is a large amount of silica on the surface of Ser‐SiO_2_@nZVI.^[^ ^64^, ^65^ ^]^

FTIR spectrum analysis is used to investigate the variations in functional groups of catalysts. Figure 1f presented the FTIR spectra of nZVI and Ser‐SiO_2_@nZVI. The band at 3430 cm^−1^ was attributed to the single‐bond OH stretching vibration or the stretching vibration of ‐OH in Si‐OH on the catalyst surface.^[^ ^62^, ^66^ ^]^ The band at 1036 cm^−1^ was related to asymmetric stretching vibration of Si‐O‐Si.^[^ ^67^ ^]^ The peak at 1632 cm^−1^ was assigned to the ‐OH bending vibrations of the surface Fe‐OH or C‐OH.^[^ ^62^ ^]^ The results further confirmed the successful coating of silica and the grafting of serine.

**Figure 2:**
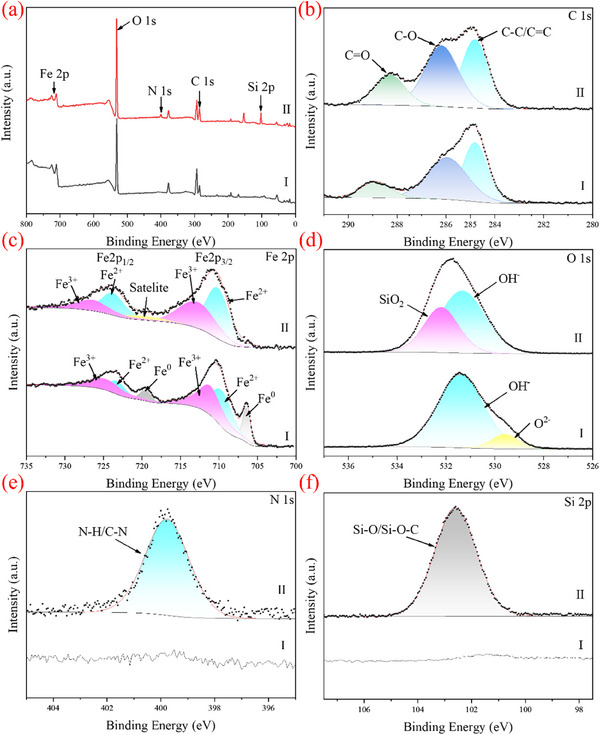
a) XPS survey spectra of nZVI (I) and Ser‐SiO_2_@nZVI (II); high‐resolution scan of b) C 1s region, c) Fe 2p region, d) O 1s region, e) N 1s region and f) Si 2p region of nZVI (I) and Ser‐SiO_2_@nZVI (II).

The XPS spectra of nZVI and Ser‐SiO_2_@nZVI were used to analyze the changes of elemental composition and chemical valence state on the surface of the catalyst before and after modification. As shown in **Figure** **2**a, the peaks of nZVI include Fe 2p and O 1s indicating the successful synthesis of nZVI. Compared with nZVI, the full spectrum of Ser‐SiO_2_@nZVI exhibits additional N 1s and Si 2p peaks, which demonstrates the successful grafting of serine and the successful coating of SiO_2_ on the surface of Fe^0^. The characteristic peaks in the C 1s spectrum can reflect the local chemical environment of carbon atoms.^[^ ^50^, ^68^ ^]^ The C 1s peaks correspond to the hydrocarbons C‐C or C‐H (284.8 eV), C‐O (286.1 eV) and O = C (288.3 eV), respectively.^[^ ^69^, ^70^ ^]^ As shown in Figure 2b, the characteristic peak intensities of C‐O and C = O increase, possibly due to the grafting of serine, leading to an increase in oxygen‐containing functional groups. Figure 2c shows three peaks at 706.4, 709.7, and 711.1 eV, corresponding to the absorption peaks of Fe (0), Fe (II) and Fe (III) peaks on the Fe 2p_3/2_ orbit.^[^ ^71^, ^72^ ^]^ As shown in Figure 2c, compared to nZVI, the Fe^0^ peak in Ser‐SiO_2_@nZVI disappears, which is because that the Fe^0^ surface has a thin iron oxide/hydroxide shell, while the coated silica makes the shell thicken, and XPS testing cannot penetrate into the internal metallic iron (Fe^0^) core.^[^ ^50^, ^73^ ^]^ At the same time, the increase of Fe^2+^ content in Ser‐SiO_2_@nZVI indicates that the silica coating is conductive to alleviating the oxidation of Fe^0^. This protective layer helps slow down the oxidation process, allowing more Fe^0^ to be present in Fe^2+^ form, which is crucial for maintaining the catalytic activity and enhancing the overall efficiency of the catalyst in oxidation reactions. The O 1s peaks of nZVI (Figure 2d) correspond to O^2−^ and OH^−^, respectively.^[^ ^74^, ^75^ ^]^ For Ser‐SiO_2_@nZVI (Figure 2d), the binding energy of O 1s at 532.2 and 531.3 eV is attributed to the oxygen in SiO_2_ and the surface hydroxyl oxygen of serine or Si‐OH, respectively. The SiO_2_ layer (5–20 nm) acts as an inert barrier, covering the Fe active sites (such as Fe^0^ and Fe^2+^) on the surface of nZVI, preventing the direct contact between oxygen molecules and the iron centers, thereby inhibiting the generation of O^2−^.^[^ ^76^, ^77^ ^]^ For nZVI, no obvious peaks were observed in the N 1s and Si 2p spectra. In the N 1s spectrum of Ser‐SiO_2_@nZVI (Figure 2e), a peak corresponding to N‐H and C‐N was observed at 399.7 eV, confirming the successful grafting of serine with amino groups onto Fe^0^.^[^ ^78^ ^]^ The Si 2p spectrum of Ser‐SiO_2_@nZVI (Figure 2f) shows a major component corresponding to the Si─O/Si─O─C bond (silicon oxide/carbon silicon oxide) at 102.6 eV.^[^ ^79^ ^]^

**Figure 3:**
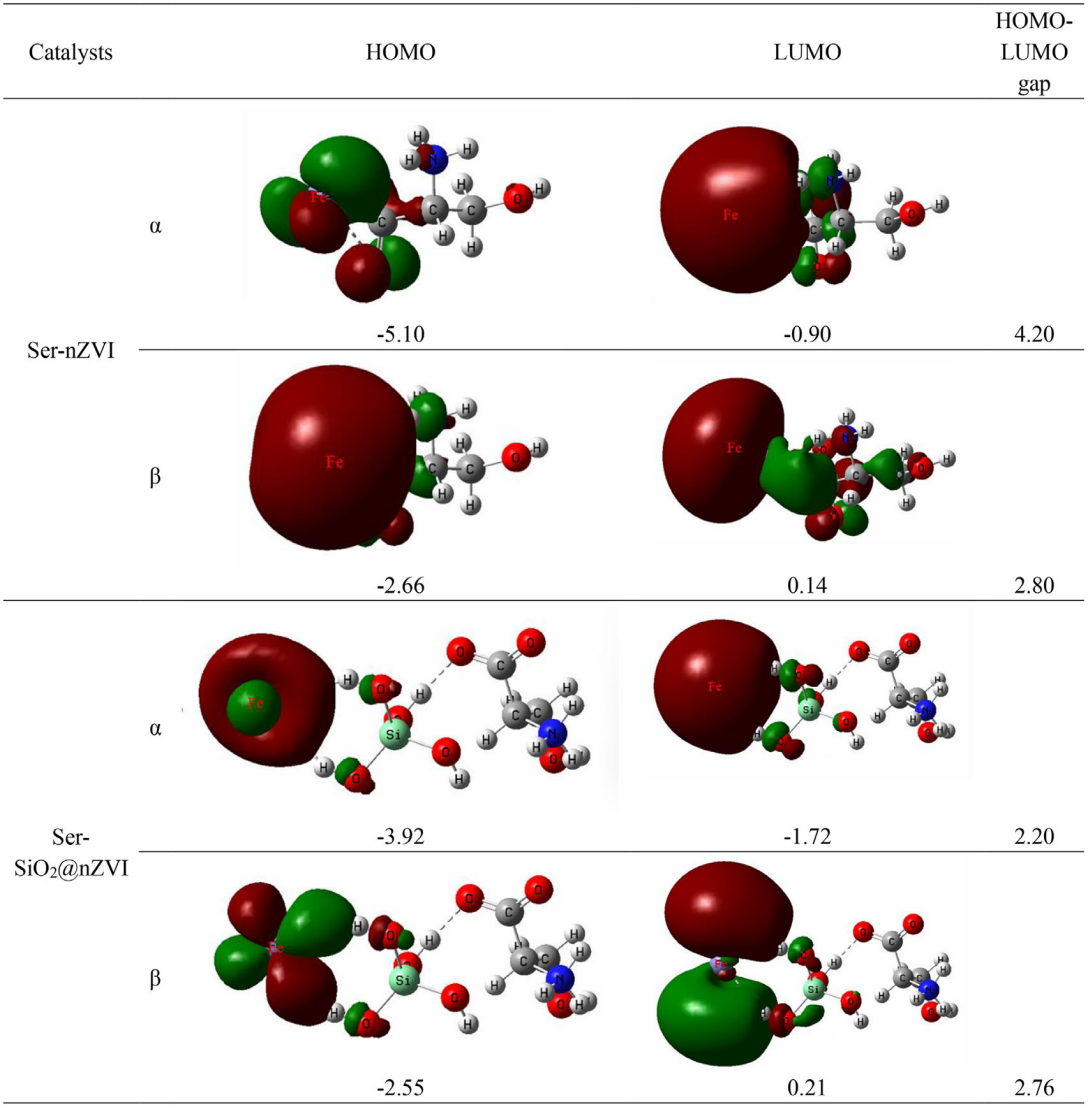
HOMO and LUMO spatial distribution and HOMO, LUMO and HOMO‐LUMO energy gap (unit: eV) of Ser‐nZVI and Ser‐SiO_2_@nZVI.

In order to further study the catalytic activities of catalysts, the HOMO/LUMO spatial analyses of Ser‐nZVI and Ser‐SiO_2_@nZVI were studied by DFT calculation (**Figure** **3**). According to the Koopmans’ theorem, the highest occupied molecular orbital (HOMO) is related to the ionization potential, and the lowest unoccupied molecular orbital (LUMO) is related to the electron affinity, thus the HOMO‐LUMO gap is generally considered as an indicator of kinetic stability and chemical reactivity to some extent.^[^ ^80^ ^]^ As shown in Figure 3, in Ser‐nZVI, the carboxyl group of serine is bonded to the Fe; in Ser‐SiO_2_@nZVI, the carboxyl group of serine is bonded to the Si─OH group, and the Si─OH group is also bonded to the Fe. The HOMO‐LUMO energy gap of Ser‐SiO_2_@nZVI is significantly smaller than that of Ser‐nZVI. The energy gap of the α orbitals decreases from 4.20 to 2.20 eV, and the energy gap of the β orbitals decreases from 2.80 eV to 2.76 eV. The results show that Ser‐SiO_2_@nZVI has smaller HOMO‐LUMO gaps and higher chemical and catalytic activity, which is consistent with the EPR test results. Due to the stronger catalytic performance of Ser‐SiO_2_@nZVI, the Ser‐SiO_2_@nZVI/H_2_O_2_ system is expected to generate more hydroxyl radicals.

In summary, the improvement in the catalytic performance of Ser‐SiO_2_@nZVI is attributed to a triple synergistic effect. Compared to nZVI, the high specific surface area and mesoporous structure of Ser‐SiO_2_@nZVI provide abundant reaction sites, while physically isolating oxygen to protect the Fe° core. The silica coating and serine grafting enhance hydrophobicity, inhibit the passivation effect caused by water molecules, and concentrate hydrophobic pollutants. The introduction of Si reduces the HOMO‐LUMO gap, improves electron transfer efficiency, and combines a strong adsorption energy to ensure effective contact between pollutants and active sites. These characteristics collectively promote the Ser‐SiO_2_@nZVI/H_2_O_2_ system to generate more •OH (verified by EPR), ultimately achieving a degradation efficiency superior to other catalysts (see Section 2.3).

### 2.2 The Adsorption of TBP/n‐dodecane by Ser‐SiO_2_@nZVI

**Figure 4:**
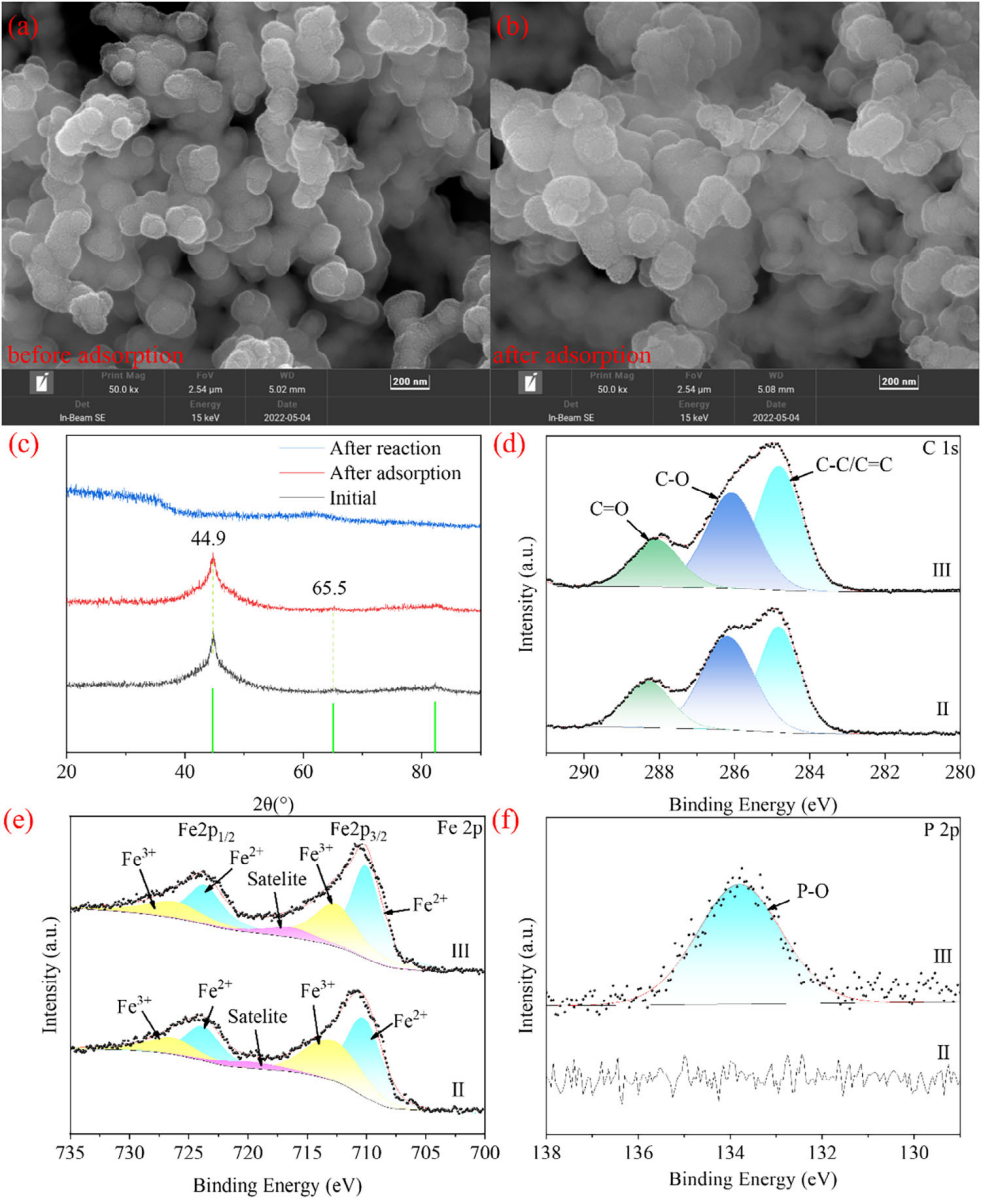
SEM images of Ser‐SiO_2_@nZVI a) before and b) after adsorption; c) XRD patterns of Ser‐SiO_2_@nZVI before and after adsorption or reaction; high‐resolution scan of d) C 1s region, e) Fe 2p region and f) P 2p region of Ser‐SiO_2_@nZVI before (II) and after (III) adsorption.

The Ser‐SiO_2_@nZVI after adsorption of TBP and n‐dodecane was characterized, and the results are shown in **Figure** **4** and Figure S4 (Supporting Information). From Figure 4a,b, it can be seen that the degree of Ser‐SiO_2_@nZVI aggregation after adsorption is significantly intensified, and the size of the material is significantly increased, probably due to the remodeling of the silica shell caused by the organic phase.^[^ ^81^ ^]^ In organic solvents, the Si─O─Si bonds in silica gradually break, leading to the dissolution of the silica shell layer. Then, as the organic solvent evaporates, SiO_2_ redeposits on the catalyst surface, causing a cycle of shell dissolution and re‐precipitation, resulting in an increase in the thickness of the core layer.^[^ ^81^, ^82^, ^83^ ^]^ The presence of P element in EDS results (Table 1) indicates that TBP is adsorbed on Ser‐SiO_2_@nZVI surface. Figure 4c and Figure S8 (Supporting Information) show that there is little change in the FTIR and XRD spectra of Ser‐SiO_2_@nZVI before and after adsorption. The adsorbed Ser‐SiO_2_@nZVI was characterized by XPS spectroscopy, and high‐resolution scanning images of C 1s, Fe 2p and P 2p regions are presented in Figure 4d–f. As shown in Figure 4d, the proportion of C─C/C = C increases after adsorption, which is speculated to be due to the adsorption of TBP and n‐dodecane onto the surface of Ser‐SiO_2_@nZVI, leading to an increase in C‐C/C = C. As shown in Figure 4e, the contents of Fe^2+^ and Fe^3+^ remain basically unchanged, indicating that the presence of a silica shell layer is beneficial for reducing the oxidation of nZVI. The silica shell serves as a protective barrier to prevent nZVI from being directly exposed to oxidative conditions, thereby helping to maintain the stability of the Fe^2+^ and Fe^3+^ ratio. This protective effect enhances the shelf life of Ser‐SiO_2_@nZVI. The appearance of P‐O peak in the high‐resolution scanning image of the P 2p region of the adsorbed Ser‐SiO_2_@nZVI (Figure 4f) also confirms that TBP is adsorbed onto the surface of Ser‐SiO_2_@nZVI.^[^ ^84^, ^85^ ^]^

**Figure 5:**
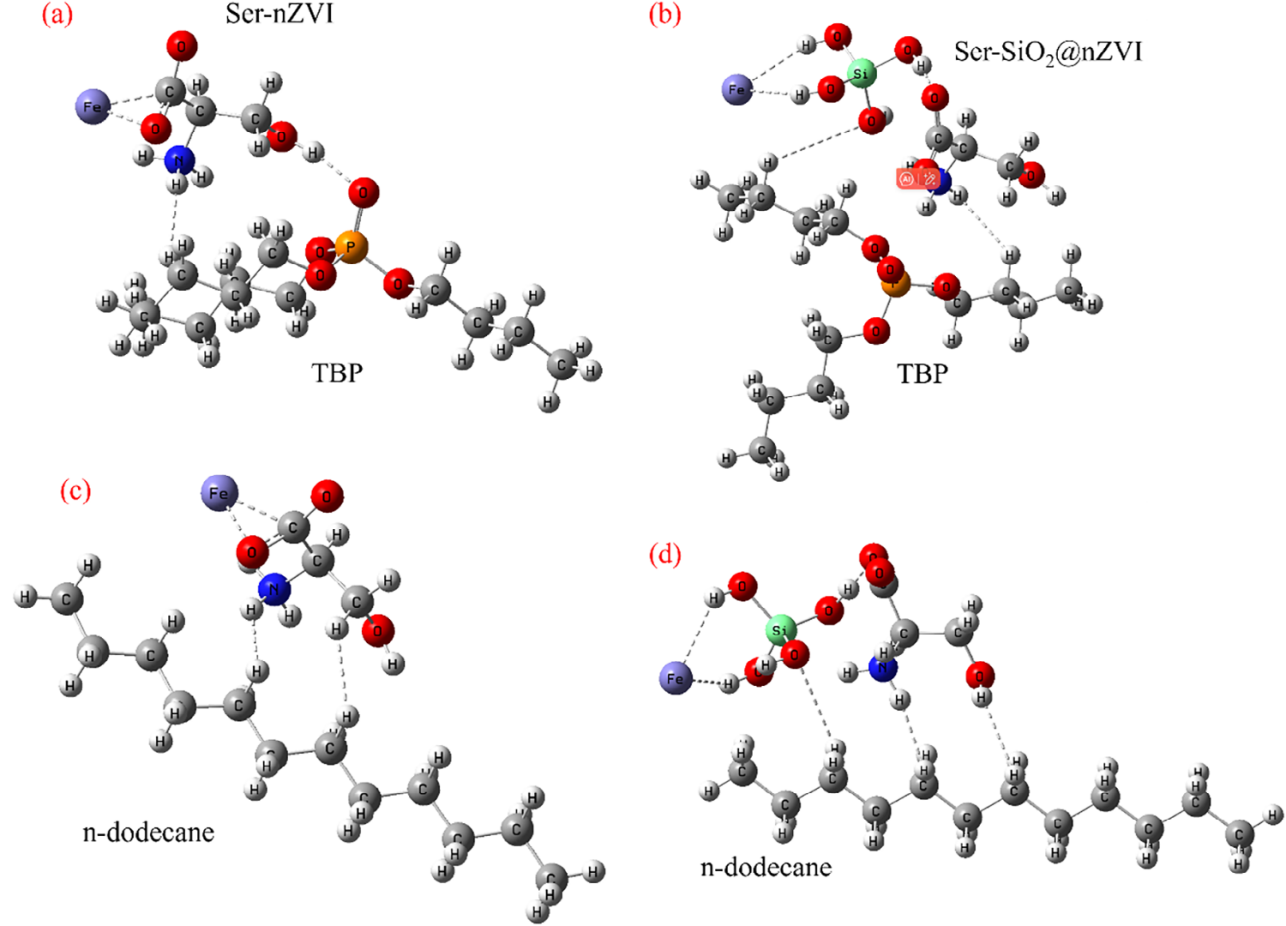
Adsorption energy calculated based on DFT simulation of TBP adsorbed by a) Ser‐nZVI and b) Ser‐SiO_2_@nZVI; DFT simulation of n‐dodecane adsorbed by c) Ser‐nZVI and d) Ser‐SiO_2_@nZVI.

The adsorption energy of TBP on the catalyst is defined as Equation (1), where *E*(TBP) is the energy of TBP, *E*(catalyst) is the energy of catalyst Ser‐nZVI or Ser‐SiO_2_@nZVI, and *E*(TBP+catalyst) is the energy of the catalyst with TBP system. The adsorption energies of n‐dodecane on Ser‐nZVI and Ser‐SiO_2_@nZVI were calculated in a similar way, and the calculation models and results are shown in **Figure** **5**. The adsorption energies of TBP on Ser‐nZVI and Ser‐SiO_2_@nZVI are −4.29 and −12.49 kcal mol^−1^, respectively, and the adsorption energies of n‐dodecane on Ser‐nZVI and Ser‐SiO_2_@nZVI are −3.54 and −11.04 kcal mol^−1^, respectively, indicating that Ser‐SiO_2_@nZVI is easier to adsorb TBP and n‐dodecane. As seen in Figure 5, Si in Ser‐SiO_2_@nZVI participates in the adsorption process, and the adsorption bonds increase, which enhances the adsorption of TBP and n‐dodecane by Ser‐SiO_2_@nZVI. The enhancement of adsorption capacity will also improve the catalytic degradation efficiency of TBP and n‐dodecane, which is consistent with the experimental results below.

(1) $$E_{\mathrm{ads}}=E\left(\mathrm{TBP}+\mathrm{catalyst}\right)-E\left(\mathrm{TBP}\right)-E\left(\mathrm{catalyst}\right)$$

### 2.3 Factors Influencing the Degradation of Organic Solvents

#### 2.3.1 Effect of Different Catalysts

**Figure 6:**
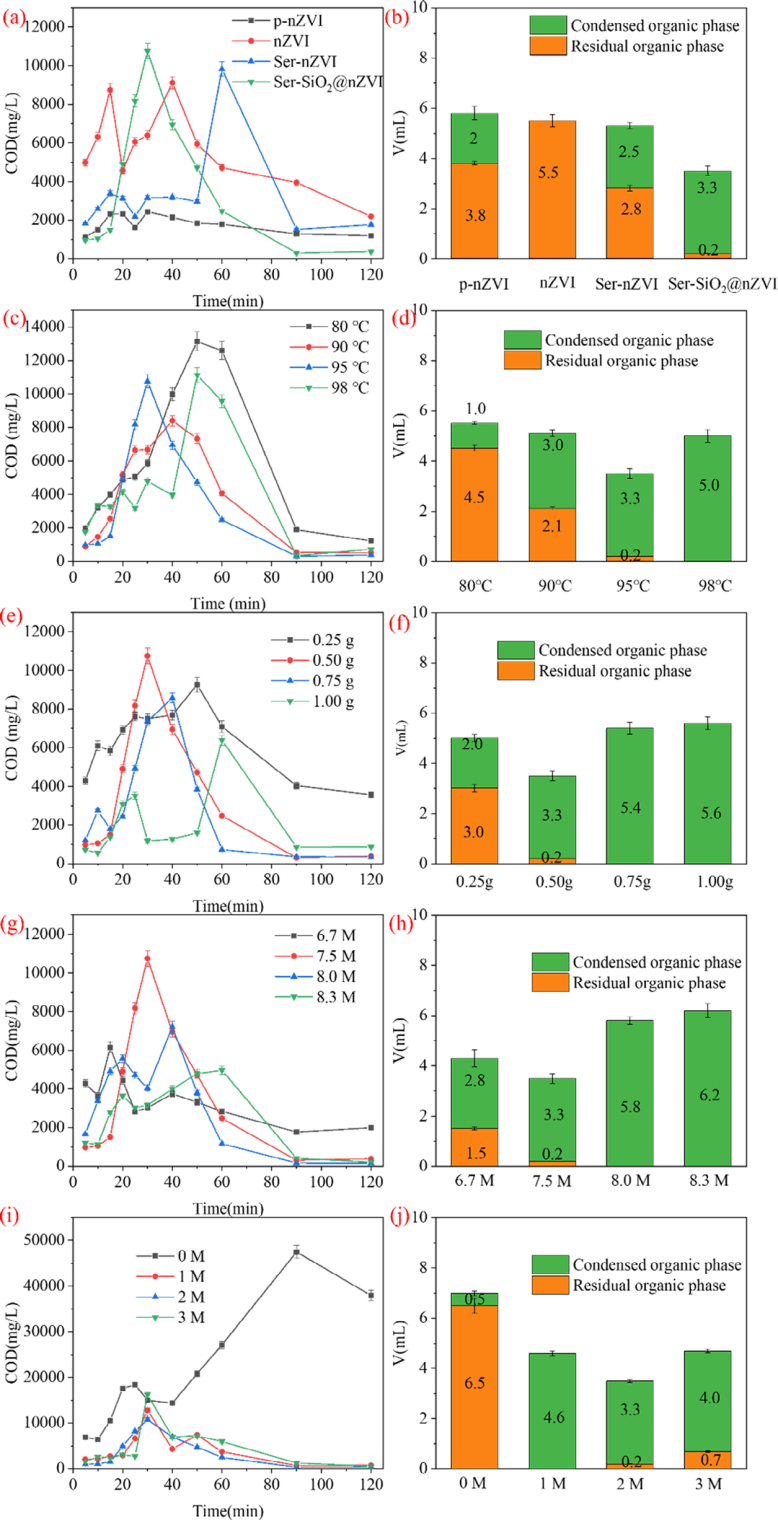
Effects of operating parameters on the degradation of TBP and n‐dodecane by heterogeneous Fenton‐like reaction: Effect of different catalysts on a) COD of aqueous solution and b) removal of organic phase; Effect of temperature on c) COD of aqueous solution and d) removal of organic phase; Effect of Ser‐SiO_2_@nZVI dose on e) COD of aqueous solution and f) removal of organic phase; Effect of H_2_O_2_ dosage on g) COD of aqueous solution and h) removal of organic phase; Effect of H_2_SO_4_ concentration on i) COD of aqueous solution and j) removal of organic phase. Except for the investigated parameter, other parameters fixed on temperature 95 °C, Ser‐SiO_2_@nZVI dose 0.5 g, 7.5 M H_2_O_2_ and 2 M H_2_SO_4_.

**Figure 7:**
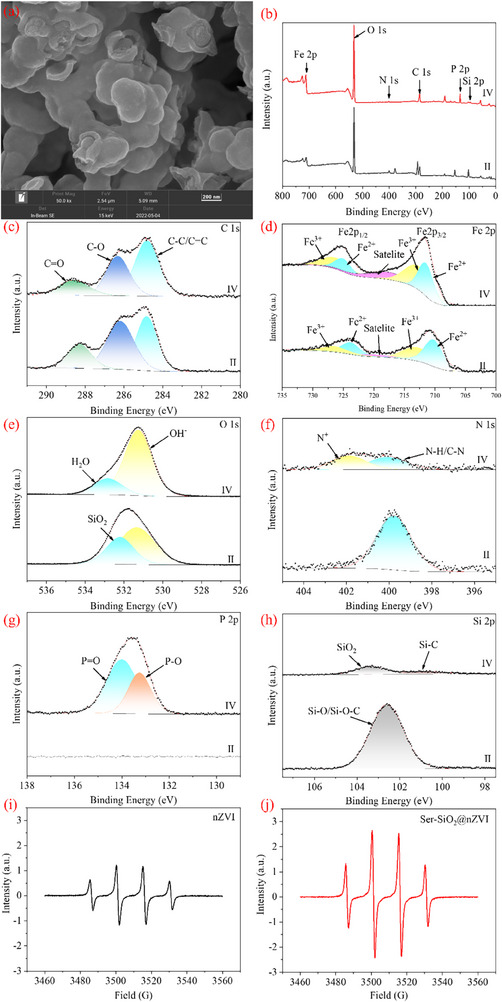
a) SEM image of Ser‐SiO_2_@nZVI after reaction; b) XPS survey spectra of Ser‐SiO_2_@nZVI before (II) and after (IV) reaction; high‐resolution scan of c) C 1s region, d) Fe 2p region, e) O 1s region, f) N 1s region, g) P 2p region and h) Si 2p region of Ser‐SiO_2_@nZVI before (II) and after (IV) reaction; ESR spectra of i) nZVI/H_2_O_2_ system and j) Ser‐SiO_2_@nZVI/H_2_O_2_ system.

**Figure** **6**a,b shows the effect of different catalysts on COD value of aqueous solution and organic phase volume with 0.5 g catalyst, 7.5 M H_2_O_2_ and 50 mL 2 M H_2_SO_4_ solution at 95 °C. Degradation experiments were conducted using purchased nanoscale zero‐valent iron (p‐nZVI), nZVI, Ser‐nVI and Ser‐SiO_2_@nZVI as the heterogeneous Fenton‐like catalyst, respectively. As shown in **Figure** **7**a, the variation trend of COD basically increased first and then decreased. The rise of COD indicated that with the progress of the reaction, the degradation of TBP and n‐dodecane produced some soluble organic matter into the aqueous solution, resulting in the increase of COD value. The decrease in COD indicated that the organic matter in the aqueous solution was further degraded and mineralized. As can be seen from Figure 6b, the organic phase after the reaction is divided into two parts, one is the remaining organic phase in the four‐mouth flask, and the other is the organic phase collected by condensation. When the reaction in the four‐mouth flask is more intense, the more organic phase will be collected by condensation. As seen in Figure 6a,b, when Ser‐SiO_2_@nZVI was used as the catalyst, the residual COD value was the lowest, the remaining organic phase was the least and the oxidation efficiency and organic phase removal rate reach 65% and 98% (Table 2), respectively, indicating that the prepared Ser‐SiO_2_@nZVI had the strongest catalytic performance, superior to p‐nZVI, nZVI and Ser‐nZVI, which is consistent with the HOMO‐LOMO gap calculated by DFT.

When nZVI and Ser‐SiO_2_@nZVI were used as catalysts, the total iron and Fe^2+^ concentrations in the reaction solution were detected, and the results are shown in Figure S9 (Supporting Information). During the reaction process, the amount of Fe released by Ser‐SiO_2_@nZVI was less than that of nZVI, and the concentration of Fe^2+^ in the solution was higher. This indicates that the presence of the silica shell layer can inhibit the excessive dissolution of iron, allowing Fe^2+^ to slowly be released into the solution. The higher concentration of Fe^2+^ is conducive to the cycling of the Fenton‐like reaction, and the slow release method can reduce the inactivation of Fe^2+^ during the reaction process, thereby ultimately improving the degradation efficiency of organic solvents.

#### 2.3.2 Effect of Temperature

Figure 6c,d shows the effect of temperature on COD value of aqueous solution and residual organic phase volume under the conditions of 0.5 g Ser‐SiO_2_@nZVI, 7.5 M H_2_O_2_ and 50 mL 2 M H_2_SO_4_ solution. It can be seen from Figure 6c that the final COD value of the degradation reaction solution gradually decreased with the increase of temperature, indicating that the Fenton reaction accelerated with the increase of temperature. As shown in Figure 6d, as the temperature increased, the remaining organic phase in the four‐necked flask decreased, and the condensed organic phase increased. This was because the Fenton reaction rate increased with increasing temperature. As the temperature increased, the evaporation of organic phase was also accelerated, and a part of organic solvents was evaporated before reacting with hydroxyl radicals (•OH).^[^ ^35^, ^86^ ^]^

The temperature change during the reaction is shown in Figure S10 (Supporting Information). The temperature first increased and then decreased, indicating that the degradation of TBP and n‐dodecane was an exothermic reaction. At the same time, it is noted that with the increase of temperature, the temperature peak gradually advanced, which was consistent with the gradual advance of the COD peak.

#### 2.3.3 Effect of Ser‐SiO_2_@nZVI Dose

The dose of Ser‐SiO_2_@nZVI significantly affected the degradation of TBP and n‐dodecane. As shown in Figure 6e,f, the effect of Ser‐SiO_2_@nZVI dose on the degradation of TBP and n‐dodecane at 95 °C with 7.5 M H_2_O_2_ and 50 mL of 2 M H_2_SO_4_ solution was studied. When the amount of Ser‐SiO_2_@nZVI increased from 0.25 to 0.5 g, the COD peak appeared earlier. After 120 min of reaction, the final COD value decreased from 3570 to 377 mg L^−1^, and the total volume of the remaining organic phase also decreased. This is because the increase of Ser‐SiO_2_@nZVI dose leads to more active sites on the catalyst surface, which is beneficial to the decomposition of H_2_O_2_ and the generation of hydroxyl radicals, improving the degradation efficiency of organic solvents.^[^ ^87^ ^]^ When the dose of Ser‐SiO_2_@nZVI was further increased to 1.0 g, the final COD value increased from 377 to 866 mg L^−1^. This is because excessive Ser‐SiO_2_@nZVI may lead to particle aggregation, and excessive Fe^2+^ will react with •OH, resulting in the reduction of •OH (Equation 2).

(2) $$Fe^{2+}+•\mathrm{OH}\to Fe^{3+}+OH^{-}$$

The influence of different doses of Ser‐SiO_2_@nZVI on the concentration of Fe^2+^ in the reaction solution was measured. As shown in Figure S11 (Supporting Information), the results indicate that when the addition of Ser‐SiO_2_@nZVI is 1.0 g, the concentration of Fe^2+^ significantly increases compared with 0.5 g. Excessive Fe^2+^ will react with •OH, and •OH is the main active substance for degrading organic solvents, thereby reducing the degradation efficiency of organic solvents and the removal rate of chemical oxygen demand (COD).

The change of the organic phase volume is also consistent with the change of COD. When the dose of Ser‐SiO_2_@nZVI was increased from 0.5 to 1.0 g, the volume of the organic phase increased and even all the organic phases were collected by condensation. This might be because as the amount of catalyst increases, the number of active sites also rises, and the reaction intensity strengthens, resulting in an increase in the evaporation volume of the organic phase or the decomposition of more TBP and n‐dodecane molecules into smaller molecular organic compounds with lower density and higher volatility. A large number of organics leaves the reaction solution by evaporation, resulting in a reduction of the organic phase degraded by Fenton reaction. In summary, when the Ser‐SiO_2_@nZVI dose is 0.5 g, the final COD value is the lowest, and the remaining organic phase volume is the smallest. In the following experiments, 0.5 g of Ser‐SiO_2_@nZVI was used as the catalyst.

#### 2.3.4 Effect of H_2_O_2_ Dosage

The effect of H_2_O_2_ was investigated by changing the dosage from 6.7 to 8.3 M while keeping the temperature at 95 °C with 0.5 g Ser‐SiO_2_@nZVI and 2 M H_2_SO_4_ solution. As shown in Figure 6g, when the H_2_O_2_ dosage was increased from 6.7 to 7.5 M, the final COD value after 120 min of reaction decreased from 1986 to 377 mg L^−1^. It shows that with the increase of the dosage of oxidant H_2_O_2_, more •OH are generated, which leads to the accelerated degradation of organic solvents. When the H_2_O_2_ dosage was further increased to 8. M, COD only slightly decreased to 151 mg L^−1^, and when the H_2_O_2_ dosage was increased to 8.3 M, COD increased to 203 mg L^−1^. It shows that further increasing the dosage of H_2_O_2_ does not significantly improve the degradation rate of organic solvents, but may lead to a decrease in the degradation rate. The reason is that excess H_2_O_2_ will react with hydroxyl radicals, as shown in Equations (3) and (4), resulting in the reduction of hydroxyl radicals, thereby hindering the removal of TBP and n‐dodecane.^[^ ^39^, ^88^ ^]^ At the same time, H_2_O_2_ will also decompose into H_2_O and O_2_ (Equation 5).^[^ ^89^ ^]^ The volume change of organic phase is shown in Figure 6h. As the dosage of hydrogen peroxide increased, the residual organic phase in the four‐necked flask decreased, and the evaporated organic phase increased. When the amount of H_2_O_2_ added was 7.5 M, the total residual amount of organic phase was the smallest, which was consistent with the change of COD.

(3) $$H_{2}O_{2}+•\mathrm{OH}\to HO_{2}•+H_{2}O$$

(4) $$HO_{2}•+•\mathrm{OH}\to H_{2}O+O_{2}$$

(5) $$2H_{2}O_{2}\to H_{2}O+O_{2}$$

#### 2.3.5 Effect of H_2_SO_4_ Concentration

The pH value is an important factor in Fenton reaction, and the concentration of H_2_SO_4_ will affect the pH of the whole system. The variation of COD and organic phase volume with the effect of H_2_SO_4_ concentration are shown in Figure 6i,j. When the concentration of H_2_SO_4_ was 0 M, the COD value first increased, then decreased and then increased, and the final value of COD was high, indicating that TBP and n‐dodecane were degraded into water‐soluble organic matter, but it was not enough for further degradation. In the four‐necked flask, there were more organic phases remaining and less organic phase condensed, indicating that the Fenton reaction was inhibited. When the concentration of H_2_SO_4_ increased to 1 M, the degradation rate of COD increased, and after further increasing the concentration of H_2_SO_4_ to 3 M, the change of COD was not obvious. As the H_2_SO_4_ concentration increased from 1 to 2 M, the remaining total organic phase decreased, indicating a further intensification of the Fenton reaction. The H_2_SO_4_ concentration increased from 2 to 3 M, and the volume of the remaining organic phase increased, indicating that Fenton reaction began to be inhibited. At lower H^+^ concentrations, the release rate of Fe^2+^ was slow, and the generation rate of hydroxyl radicals (•OH) on the surface of Ser‐SiO_2_@nZVI was limited, which hindered the continuation of free radical chain reactions. Conversely, excessive H^+^ concentration could neutralize the •OH radicals, negatively affecting the degradation of organic solvents.

### 2.4 Nuclide Equilibrium Experiment

Radiation protection is extremely important in the treatment of radioactive organic waste. Therefore, it is necessary to study the distribution of nuclides in the system of the Fenton‐like oxidation of radioactive waste organic solvents, and to investigate whether there are differences in the degradation of organic solvents with or without nuclides. Nd^3+^ and Ce^4+^ were used to simulate trivalent and tetravalent nuclides, respectively, which may have properties closer to real radioactive nuclides. After Fenton‐like degradation of organic solvents containing Nd^3+^, the content of Nd^3+^ in the aqueous phase and oil phase of residual liquid was 5917.52 and 0.054 µg, and the content of Nd^3+^ in water phase and oil phase of condensate oil was 3.45 and 0.039 µg, respectively. The content of Ce^4+^ in the aqueous and oil phases of the residual liquid was 5839.65 and 0.13 µg, and the content of Ce^4+^ in the water and oil phases of condensate oil was 4.3 and 0.23 µg, respectively. This means that over 99% of the nuclides remained in the residual liquid, while the condensate oil contained almost no nuclides. In addition, the degradation experiments of TBP/n‐dodecane obtained after the extraction of Sr and U were also conducted. The results show that the U content in the aqueous and oil phases of the residual liquid was 11485.9 and 183.6 µg, and the U content in the water and oil phases of the condensate liquid was 841.8 and 60.2 µg, respectively. More than 90% of the U element and over 99% of the Sr nuclide still remained in the four necked flask.

Figure S12a,b (Supporting Information) represent the effects of nuclides on the degradation of organic solvents and the removal of oil phase volume, respectively. As can be seen from the figure, the degradation of organic solvents with and without nuclides was almost the same, and the presence of nuclides had little effect on the degradation of organic solvents. This verified the effectiveness, feasibility and safety of the heterogeneous Fenton‐like system for the degradation of radioactive organic solvents.

### 2.5 Reaction Mechanism

The catalyst Ser‐SiO_2_@nZVI for the degradation of TBP and n‐dodecane after 5 min was characterized, and the results are shown in Figure 7 and Figure S5 (Supporting Information). From the SEM results (Figure 7a), it can be clearly seen that the shell of SiO_2_ was destroyed, resulting in the exposure of Fe^0^ in the center. EDS results (Table 1) showed that Fe element decreased and O element increased, indicating that Fe^0^ was oxidized; the increase of P element suggested that TBP was oxidized and degraded on the surface of Ser‐SiO_2_@nZVI. This is consistent with the disappearance of SiO_2_ peak in the high‐resolution scanning image of O 1s region and the decrease of peak intensity in the high‐resolution scanning image of Si 2p region.^[^ ^90^, ^91^ ^]^ From the high‐resolution scanning image of the C 1s region (Figure 7c), it can be seen that the content of C‐C/C = C increases because TBP and n‐dodecane were adsorbed onto the surface of Ser‐SiO_2_@nZVI, promoting the degradation of organic solvents. This is consistent with the appearance of P‐O and P = O peaks in Figure 7e.^[^ ^84^, ^85^ ^]^ From the high‐resolution scan image of the Fe 2p region (Figure 7d), it can be seen that after 5 minutes of reaction, the concentration ratio of Fe^3+^ increases, and the concentration ratio of Fe^2+^ decreases. The SiO_2_ shell, which typically acts as a protective barrier, may have been compromised under the reaction conditions, leading to a greater exposure of Fe^0^ and Fe^2+^ to oxidative species like H_2_O_2_. This results in the oxidation of Fe^2+^ to Fe^3+^, thus altering the iron oxidation state distribution. As can be seen from Figure 1f, the peak at 1342 cm^−1^ is associated with nZVI. However, in Figure S8 (Supporting Information), this peak disappeared after reaction, indicating that nZVI gradually decreased during degradation, which is consistent with the results of XPS. In the high‐resolution scanning image of N1s region (Figure 7f), it can be seen that the N‐H/C‐N peak decreases and the protonation amine (N^+^) appears, indicating the protonation of ‐NH_2_ in acid solution.^[^ ^92^ ^]^

**Table 2:** The final COD value, oxidation efficiency and organic phase removal rate of organic solvents by different catalysts under the optimal conditions.

Type of catalyst	Final COD value [mg L^−1^]	oxidation efficiency [%]	organic phase removal rate [%]
p‐nZVI	1191.43	42	62
nZVI	2191.66	45	45
Ser‐nZVI	1764.73	47	72
Ser‐SiO_2_@nZVI	377.2	65	98

In order to further verify the generation of •OH in nZVI/H_2_O_2_ system and Ser‐SiO_2_@nZVI/H_2_O_2_ system, EPR tests were conducted under the same conditions. The hydroxyl radicals produced in the systems were captured by DMPO as the trapping agent, which were quantitatively analyzed. The ESR spectrum of DMPO‐•OH exhibits four typical characteristic peaks with a ratio of 1:2:2:1,^[^ ^93^ ^]^ as shown in Figure 7i,j. Since the intensity of the peaks corresponds to the generation of •OH, it can be seen from the figures that the Ser‐SiO_2_@nZVI/H_2_O_2_ system produces more hydroxyl radicals, which is consistent with the above experimental results.^[^ ^94^ ^]^ Under the condition of silica coating, reactions such as those shown in Equations (6) and (7) can occur, leading to the production of OH• and HO_2_• and the generation of more hydroxyl radicals, which enhances further degradation of pollutants.^[^ ^95^, ^96^ ^]^

(6) $$\mathrm{Si}O_{2}-Fe^{3+}+H_{2}O_{2}\to \mathrm{Si}O_{2}-Fe^{2+}+HO_{2}•+H^{+}$$

(7) $$\mathrm{Si}O_{2}-Fe^{2+}+H_{2}O_{2}\;\to \mathrm{Si}O_{2}-Fe^{3+}+HO^{-}+\mathrm{OH}•$$

**Figure 8:**
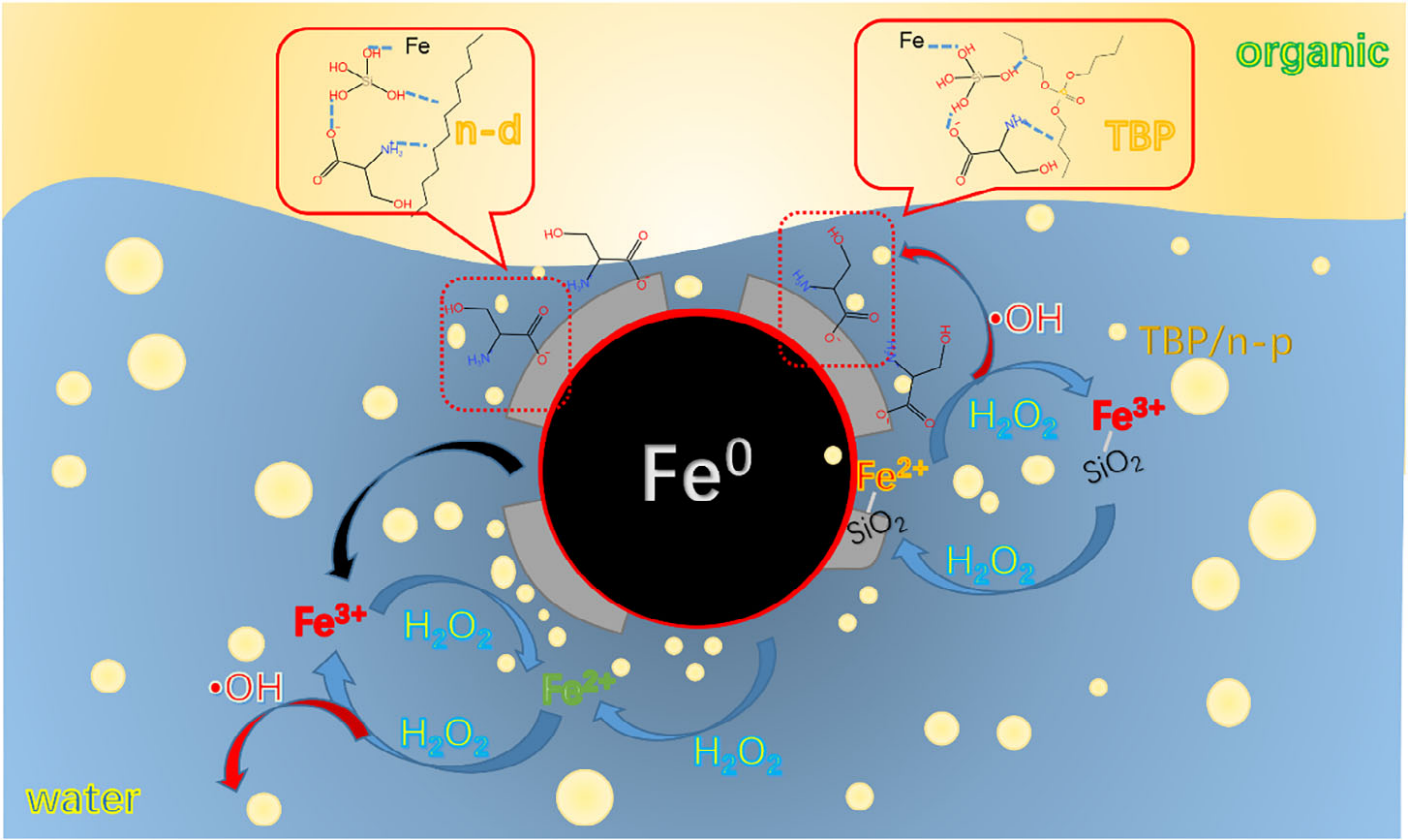
Adsorption and catalytic degradation mechanism of organic solvents by Ser‐SiO_2_@nZVI/H_2_O_2_ system.

In summary, compared with Ser‐nZVI, the catalytic performance of Ser‐SiO_2_@nZVI as a heterogeneous Fenton‐like catalyst is enhanced. As shown in the mechanism diagram of **Figure** **8**, the SiO_2_ coating can fix more serine molecules on the surface of nZVI, thereby enhancing the adsorption performance of the catalyst for TBP and n‐ dodecane. The ‐OH groups on the surface of the SiO_2_ coating can also interact with the side chain of TBP or the hydrogen atoms on the C chain of n‐dodecane, strengthening the adsorption performance of Ser‐SiO_2_@nZVI for TBP and n‐dodecane. This interaction enables more TBP and n‐dodecane to be adsorbed on the surface of Ser‐SiO_2_@nZVI. At the same time, the presence of the SiO_2_ shell helps protect nZVI from rapid erosion or dissolution by acidic solutions, reduces the oxidation of Fe^0^, and maintains a higher Fe^2+^ ratio rather than Fe^3+^ ratio. This is crucial for maintaining the catalytic activity of Ser‐SiO_2_@nZVI in the oxidation reaction and improving its overall efficiency. More Fe^2+^ will increase the generation of •OH, and •OH has high reactivity and plays an important role in the degradation of organic solvents. Therefore, the improvement of adsorption performance and the stabilization of Fe^2+^ work together to enhance the degradation efficiency of organic solvents and exhibit better performance in the catalytic process.

## 3 Conclusion

This study successfully synthesized Ser‐SiO_2_@nZVI catalyst, which was used for efficient degradation of radioactive organic solvents (TBP/n‐dodecane mixed system) generated during nuclear fuel reprocessing. Under the optimal reaction conditions of temperature 95 °C, 0.5 g Ser‐SiO_2_@nZVI catalyst, 7.5 M H_2_O_2_ oxidant and 2.0 M H_2_SO_4_, the oxidation efficiency of organic solvents could reach more than 60%, and the removal efficiency of organic solvents could reach more than 95%, which was much higher than that of unmodified nZVI. Through the dual modification of serine grafting and silica coating, the catalytic stability and adsorption performance of nZVI were significantly improved. The SiO_2_ shell physically isolated oxygen, protected the Fe° core, inhibited the rapid oxidation of nZVI, and released Fe^2+^ to continuously generate hydroxyl radicals. The narrowing of the HOMO‐LUMO energy gap promoted electron transfer and increased the yield of •OH. The synergistic effect of surface functional groups (carboxyl groups of serine, amino groups, and hydroxyl groups of SiO_2_) enhanced the adsorption of organic solvents. Nuclear isotope simulation experiments showed that over 90% of the isotopes were fixed in the reaction vessel, confirming the safety of treating radioactive organic solvents. This catalyst operates at low temperatures and normal pressures, avoiding the secondary pollution risk, and can be directly applied to the treatment of waste solvents at nuclear power plants, providing a universal solution for the treatment of hydrophobic organic pollutants.

## 4 Experimental Section

### Reagents

Ferrous sulfate heptahydrate (FeSO_4_•7H_2_O), tetraethyl orthosilicate (TEOS, ≥98.0%), ethanol(C_2_H_6_O) and sulfuric acid (H_2_SO_4_) were supplied by Sinopharm Chemical Reagent Co., Ltd. (Shanghai, China). DL‐Serine(C_3_H_7_NO_3_) and n‐Dodecane (C_12_H_26_) were purchased from Aladdin Biotechnology Co., Ltd. (Shanghai, China). Potassium borohydride (KBH_4_) was purchased from Kemiou Chemical Reagent Co., Ltd. (Tianjin, China). Hydrogen peroxide (H_2_O_2_) (30%, v/v) was obtained from Kelong Chemical Co., Ltd. (Chengdu, China). Tributyl phosphate (TBP) was supplied by Macklin Biochemical Co., Ltd. (Shanghai, China). Argon (Ar) was supplied by Wuhan ZXRY Gas Co, Ltd. (Wuhan, China). All chemical solutions and reagents were analytical and used without further purification.

### Preparation and Characterization of Catalysts

The nZVI used in this experiment was prepared according to the previous method in our laboratory.^[^ ^35^ ^]^ To increase the yield of nZVI, the concentrations of FeSO_4_•7H_2_O and KBH_4_ were increased by 10 times. The catalyst was synthesized in a 500 mL four‐necked flask under Ar protection at ambient temperature. 100 mL of 0.4 M FeSO_4_•7H_2_O solution was placed in the reaction vessel while stirring vigorously. Then 100 mL of 2 M KBH_4_ solution was added dropwise to the reaction vessel at a flow rate of 2 mL/min. After the dropwise addition was completed, stirring was continued for 1 h to ensure sufficient reaction. The synthesized nZVI particles were washed several times with ionized water and ethanol, respectively. Finally, nZVI was dried in a freeze drier. Silica coated iron nanoparticle (SiO_2_@nZVI) was synthesized by the Stöber method,^[^ ^49^ ^]^ that is, 0.01 mol TEOS was added to the nZVI solution and stirred for 4 h. Then, Ser‐SiO_2_@nZVI particles were obtained by adding 50 mL of 0.5 g L^−1^ serine solution to SiO_2_@nZVI solution and stirring for 12 h. Ser‐nZVI particles were obtained by adding 50 mL of 0.5 g L^−1^ serine solution to the nZVI solution with stirring for 12 h.

The prepared nZVI, Ser‐nZVI and Ser‐SiO_2_@nZVI were characterized by scanning electronic microscopy with X‐ray energy dispersive spectroscopy (SEM‐EDS, TESCAN MIRA LMS, Czech Republic) to obtain the surface morphology, particle size and element distribution of the particles. N_2_ adsorption and desorption isotherms were measured using a Brunauer‐Emmett‐Teller (BET) sorptometer (Micromeritics ASAP 2460) to investigate the specific surface area and pore size distribution of catalysts. X‐ray diffraction (XRD, Bruker D8 advance, 40 kV and 40 mA, Cu Kα) with a scanning range from 5° to 90° at a speed of 2°/min and Fourier transform infrared spectrometer (FTIR, Thermo Scientific Nicolet 6700) were determined. The nanoparticles of nZVI and Ser‐SiO_2_@nZVI were characterized by X‐ray photoelectron spectrometer (XPS, Thermo Scientific K‐Alpha+). The water contact angles were measured on a DSA100 contact angle tester. The zeta potential at pH 2, 5, 7 and 11 was determined by a Malvern Zetasizer Nano ZS90 instrument.

### Experimental Procedure

The adsorption experiments in this study were carried out in a four‐necked flask. First, Ser‐SiO_2_@nZVI, 3 mL TBP and 7 mL n‐dodecane were added to the flask. Then, vigorous stirring was performed to make Ser‐SiO_2_@nZVI fully adsorb TBP and n‐dodecane. Finally, the adsorbed Ser‐SiO_2_@nZVI was characterized and analyzed.

The degradation experiments were also carried out in a four‐necked flask (500 mL) placed in a constant temperature water bath, and the organic phase evaporated was collected using a condensation device. First, the catalyst, 3 mL TBP and 7 mL n‐dodecane were added to the flask. Then, the reaction was initiated by dripping sulfuric acid solution and hydrogen peroxide through a peristaltic pump. The reaction solution was stirred using magnetic stirring to make the mixture uniform. During the reaction, samples were taken at regular intervals to measure the chemical oxygen demand (COD) of the aqueous phase. After the reaction was completed, the remaining organic phase in the four‐necked flask and the organic phase collected by the condensing device were measured. The ratio of the volume of organic solvents reduced by Fenton‐like oxidation to the total volume was calculated as the oxidation efficiency (%) of organic solvents. Additionally, since the presence of radioactive nuclides in the organic solvents evaporated by condensation was negligible, the ratio of the volume of organic solvents removed by both Fenton‐like oxidation and condensation to the total volume was calculated as the removal efficiency (%) of organic solvents.

### Analytical Methods

With the progress of degradation, TBP and n‐dodecane were gradually degraded, and the degradation products dissolved in water, causing the change of COD in the aqueous phase. According to the Environmental Protection Industry Standard (HJ/T 399‐2007) of the People's Republic of China, the COD values of the samples in the degradation experiment were measured by rapid digestion spectrophotometric method. The concentrations of Nd^3+^ and Ce^4+^ were determined by inductively coupled plasma‐mass spectrometry (ICP‐MS, Agilent 7800, USA). The concentration of Sr was measured using an atomic absorption spectrophotometer (AAS, WFX‐220 A, Raleigh, China). According to the Environmental Protection Industry Standard of the People's Republic of China (HJ 840–2017), the content of the U element in the experiment was extracted using the analytical method for trace uranium in environmental samples. The Bruker EPR A300 electron paramagnetic resonance spectrometer (EPR, Germany) was used to detect •OH captured by 5,5‐dimethyl‐l‐pyrroline‐N‐oxide (DMPO).

### DFT Calculations

All DFT calculations were created using Gaussian View 5.0 and calculated using Gaussian 16 software.^[^ ^97^ ^]^ The hybrid Becke‐3 Lee–Yang–Parr (B3LYP) density functional theory method with the 6–31g** basis set was used for structural optimization and frequency calculation to ensure that the calculation results were close to reasonable values.^[^ ^98^ ^]^ For models involving Fe, the LANL2DZ pseudopotential and the corresponding basis sets were applied. In addition, the SMD solvation model was added to simulate the water environment.^[^ ^99^ ^]^

### Statistical analysis

All experimental degradation trials, including those investigating the effects of catalyst type, temperature, catalyst dosage, H_2_O_2_ dosage, and H_2_SO_4_ concentration, were conducted in at least triplicate to ensure data reliability and reproducibility. Results were presented as the mean value ± standard deviation (SD).

## Conflict of Interest

The authors declare no conflict of interest.

## Supporting information



Supporting Information

## Data Availability

The data that support the findings of this study are available from the corresponding author upon reasonable request.

## References

[1] J. Pearson , O. Jan , G. Miller , M. Nilsson , Procedia Chemistry 2012, 7, 334.

[2] A. P. Paiva , P. Malik , J. Radioanal. Nucl. Chem. 2004, 261, 485.

[3] Q. Sun , L. Jiang , L. Gong , J. H. Sun , J. Hazard. Mater. 2016, 314, 230. 27136728 10.1016/j.jhazmat.2016.04.030

[4] L. Leay , K. Tucker , A. Del Regno , S. L. M. Schroeder , C. A. Sharrad , A. J. Masters , Mol. Phys. 2014, 112, 2203.

[5] S. V. Jala , S. V. Sreekantan , S. Mahadevan , H. Seshadri , A. B. Mandal , J. Therm. Anal. Calorim. 2016, 124, 1525.

[6] A. Dodi , G. Verda , J. Chromatogr. A 2001, 920, 275. 11453010 10.1016/s0021-9673(01)00834-2

[7] L. Borges Silverio , W. D. Q. Lamas , Energy Policy 2011, 39, 281.

[8] V. Babain , M. Alyapyshev , I. Voronaev , L. Tkachenko , E. Kenf , Solvent Extr. Ion Exch. 2021, 39, 255.

[9] S. V. Sreekantan , S. Mahadevan , S. V. K. Jala , H. Seshadri , A. B. Mandal , Org. Process Res. Dev. 2014, 18, 1821.

[10] S. C. Tripathi , K. K. Gupta , M. Bindu , P. M. Gandhi , J. Radioanal. Nucl. Chem. 2013, 295, 657.

[11] A. Wright , P. Paviet‐Hartmann , Separation Science and Technology 2010, 45, 1753.

[12] J. Li , L. Chen , J. Wang , Progress in Nuclear Energy 2021, 141, 103957.

[13] E. A. Barinova , M. N. Diordii , O. K. Karlina , Y. V. Karlin , Atomic Energy 2016, 120, 426.

[14] S. Ma , C. Park , S.‐H. Min , M. Kim , B. H. Hong , Journal of the Korean Physical Society 2022, 81, 221.

[15] S. V. Kulkarni , V. L. Markad , J. S. Melo , S. F. D'Souza , K. M. Kodam , S3. Applied Microbiology and Biotechnology 2014, 98, 919. 23644771 10.1007/s00253-013-4938-2

[16] S. S. Rangu , B. Muralidharan , S. C. Tripathi , S. K. Apte , Appl. Microbiol. Biotechnol. 2014, 98, 2289. 23963271 10.1007/s00253-013-5158-5

[17] B. Y. Min , Y. J. Lee , G. S. Yun , K. W. Lee , J. K. Moon , Ann. Nucl. Energy 2015, 80, 47.

[18] S. A. Walling , M. N. Kauffmann , L. J. Gardner , D. J. Bailey , M. C. Stennett , C. L. Corkhill , N. C. Hyatt , J. Hazard. Mater. 2021, 401, 123764. 33113733 10.1016/j.jhazmat.2020.123764

[19] S. B. Eskander , S. M. Abdel Aziz , H. El‐Didamony , M. I. Sayed , J. Hazard. Mater. 2011, 190, 969. 21536381 10.1016/j.jhazmat.2011.04.036

[20] W. Zhang , J. Li , J. Wang , Journal of Nuclear Science and Technology 2015, 52, 1362.

[21] T. D. Chaudhari , S. Eapen , M. H. Fulekar , Journal of Toxicology and Environmental Health Sciences 2009, 1, 001.

[22] R. A. P. Thomas , L. E. Macaskie , Appl. Microbiol. Biotechnol. 1998, 49, 202. 9534259 10.1007/s002530051159

[23] T. D. Chaudhari , J. S. Melo , M. H. Fulekar , S. F. D'Souza , International Biodeterioration & Biodegradation 2012, 74, 87.

[24] M. Mabrouk , F. Lemont , J. M. Baronnet , J. Phys.: Conf. Ser. 2012, 406, 012002.

[25] J. Li , J. Wang , J. Hazard. Mater. 2006, 135, 443. 16388899 10.1016/j.jhazmat.2005.11.053

[26] S. Wang , G. Yu , J. Wang , Chemosphere 2023, 317, 137889. 36657574 10.1016/j.chemosphere.2023.137889

[27] T. D'halluin , C. Lepeytre , A. Leydier , C. Julcour , Environ. Technol. 2021, 42, 4247. 32249685 10.1080/09593330.2020.1751731

[28] J. J. Ducoste , S. M. Alpert , Water Quality Research Journal 2014, 50, 4.

[29] J. Wang , S. Wang , Chem. Eng. J. 2020, 401, 126158.

[30] J. L. Wang , L. J. Xu , Crit. Rev. Environ. Sci. Technol. 2012, 42, 251.

[31] C. Wang , G. Yu , J. Wang , Progress in Nuclear Energy 2020, 130, 103563.

[32] L. Xu , X. Meng , M. Li , W. Li , Z. Sui , J. Wang , J. Yang , Chem. Eng. J. 2019, 361, 1511.

[33] Z. Wan , L. Xu , J. Wang , Chem. Eng. J. 2016, 284, 733.

[34] L. J. Liu , B. L. Tang , Journal of Nuclear and Radiochemistry 2005, 27, 1.

[35] X. Yang , G. Yu , L. Xu , J. Wang , Chemosphere 2022, 292, 133449. 34973247 10.1016/j.chemosphere.2021.133449

[36] D. V. Kerkez , D. D. Tomašević , G. Kozma , M. R. Bečelić‐Tomin , M. D. Prica , S. D. Rončević , Á. Kukovecz , B. D. Dalmacija , Z. Kónya , J. Taiwan Inst. Chem. Eng. 2014, 45, 2451.

[37] A. N. Soon , B. H. Hameed , Desalination 2011, 269, 1.

[38] P. Mondal , A. Anweshan , M. K. Purkait , Chemosphere 2020, 259, 127509. 32645598 10.1016/j.chemosphere.2020.127509

[39] L. Xu , J. Wang , J. Hazard. Mater. 2011, 186, 256. 21109349 10.1016/j.jhazmat.2010.10.116

[40] J. Sun , J. Yang , Y. Liu , M. Guo , Q. Wen , W. Sun , J. Yao , Y. Li , F. Jiang , Water Res. 2019, 157, 621. 31004978 10.1016/j.watres.2019.03.066

[41] H. Lv , H. Niu , X. Zhao , Y. Cai , F. Wu , Appl. Catal., B 2021, 286, 119940.

[42] B. Kumari , S. Dutta , Journal of Water Process Engineering 2020, 37, 101370.

[43] Z. H. Diao , W. Qian , Z. X. Lei , L. J. Kong , J. J. Du , H. Liu , J. W. Yang , S. Y. Pu , Sci. Total Environ. 2019, 660, 541. 30641381 10.1016/j.scitotenv.2019.01.037

[44] Q. Ma , W. Teng , Y. Sun , Y. Chen , Y. Xue , X. Chen , C. Zhang , H. Zhang , J. Fan , Y. Qiu , R. Fu , Sci. Total Environ. 2022, 827, 154329. 35257767 10.1016/j.scitotenv.2022.154329

[45] Z. Guan , J. Wan , Y. Ma , Y. Wang , Y. Shu , Bioinorg. Chem. Appl. 2015, 2015, 548961. 26060427 10.1155/2015/548961 PMC4427803

[46] S. Chen , J. Bedia , H. Li , L. Y. Ren , F. Naluswata , C. Belver , Chem. Eng. J. 2018, 343, 619.

[47] J. Wan , J. Wan , Y. Ma , M. Huang , Y. Wang , R. Ren , Chem. Eng. J. 2013, 221, 300.

[48] H. Lu , J. Dong , M. Zhang , W. Hu , C. Wen , C. Yang , Y. Wu , Colloids Surf. A 2018, 558, 271.

[49] Z. Guan , Y. Shu , Y. Ma , J. Wan , Colloids Surf. A 2015, 482, 18.

[50] X. Yang , F. Ming , J. Wang , L. Xu , Journal of Environmental Sciences 2024, 135, 296. 10.1016/j.jes.2022.11.013 37778805

[51] N. Tsolekile , S. Parani , R. Maluleke , O. Joubert , M. C. Matoetoe , S. P. Songca , O. S. Oluwafemi , Dyes Pigm. 2021, 185, 108960.

[52] H. Zhang , J. Wang , Y. Zeng , G. Wang , S. Han , Z. Yang , B. Li , X. Wang , J. Gao , L. Zheng , X. Liu , Z. Huo , R. Yu , Phys. Lett. A 2020, 384, 126600.

[53] H. Zhao , Y. Yang , X. Shu , Y. Wang , Q. Ran , Adv. Colloid Interface Sci. 2018, 256, 230. 29656761 10.1016/j.cis.2018.04.003

[54] J. Peng , Y. Zhao , X. Wang , X. Zeng , J. Wang , S. Hou , Mater. Today Commun. 2024, 40, 109780.

[55] J. Fang , K. Xu , A. Liu , Y. Xue , L. Tie , Z. Deng , R. Qiu , W. X. Zhang , Environ. Sci.: Nano 2024, 11, 1915.

[56] N. Li , J. Zhu , X. Ma , Q. Zha , C. Song , AIChE J. 2013, 59, 1236.

[57] Y. Yang , L. Xu , W. Li , W. Fan , S. Song , J. Yang , Appl. Catal., B 2019, 259, 118057.

[58] L. Zhou , K. Wang , Y. Yi , Z. Fang , Journal of Environmental Management 2023, 326, 116775. 36402015 10.1016/j.jenvman.2022.116775

[59] C. Ling , S. Wu , T. Dong , H. Dong , Z. Wang , Y. Pan , J. Han , J. Hazard. Mater. 2022, 423, 127082. 34488104 10.1016/j.jhazmat.2021.127082

[60] R. Saad , S. Thiboutot , G. Ampleman , W. Dashan , J. Hawari , Chemosphere 2010, 81, 853. 20801482 10.1016/j.chemosphere.2010.08.012

[61] X. Sun , H. Yu , D. Zheng , X. Wang , J. Li , L. Wang , Appl. Surf. Sci. 2013, 279, 1.

[62] Y. Huang , W. Zhang , M. Zhang , X. Zhang , Y. Zhao , Chem. Eng. J. 2018, 338, 369.

[63] Y. Li , X. Cai , J. Guo , S. Zhou , P. Na , Appl. Surf. Sci. 2015, 324, 179.

[64] F. S. D. Santos , F. R. Lago , L. Yokoyama , F. V. Fonseca , J. Mater. Res. Technol. 2017, 6, 178.

[65] M. Kokunešoski , J. Gulicovski , B. Matović , M. Logar , S. K. Milonjić , B. Babić , Mater. Chem. Phys. 2010, 124, 1248.

[66] H. Lu , C. Wen , S. Gao , Y. Dong , M. Zhang , B. Li , W. Hu , J. Dong , Colloids Surf. A 2018, 553, 28.

[67] S. Li , S. Li , N. Wen , D. Wei , Y. Zhang , Sep. Purif. Technol. 2021, 265, 118341.

[68] A. Mocellin , A. H. D. A. Gomes , O. C. Araújo , A. N. de Brito , O. Björneholm , J. Phys. Chem. B 2017, 121, 4220. 28358197 10.1021/acs.jpcb.7b02174

[69] F. Sun , Y. Zhu , X. Liu , Z. Chi , Environ. Sci. Pollut. Res. 2023, 30, 27560. 10.1007/s11356-022-24226-8 36385336

[70] Y. Ma , X. Lv , Q. I. Yang , Y. Wang , X. Chen , Appl. Catal., A 2017, 542, 252.

[71] Y. Wang , Y. Gong , N. Lin , L. Yu , B. Du , X. Zhang , J. Colloid Interface Sci. 2022, 606, 941. 34487941 10.1016/j.jcis.2021.08.075

[72] Z. Qiu , Q. Tian , T. Zhang , D. Yang , F. Qiu , Sep. Purif. Technol. 2020, 230, 115847.

[73] W. Yan , A. A. Herzing , X. Q. Li , C. J. Kiely , W. X. Zhang , Environ. Sci. Technol. 2010, 44, 4288. 20446741 10.1021/es100051q

[74] H. Chen , H. Xie , J. Zhou , Y. Tao , Y. Zhang , Q. Zheng , Y. Wang , Water Science and Technology 2019, 80, 1076. 31799951 10.2166/wst.2019.358

[75] Y. Wu , Q. Yue , Y. Gao , Z. Ren , B. Gao , Journal of Environmental Sciences 2018, 69, 173. 10.1016/j.jes.2017.10.006 29941253

[76] H. Liu , P. Li , H. Yu , T. Zhang , F. Qiu , Chem. Eng. Res. Des. 2019, 151, 242.

[77] X. Sun , Y. Yan , J. Li , W. Han , L. Wang , J. Hazard. Mater. 2014, 266, 26. 24374562 10.1016/j.jhazmat.2013.12.001

[78] L. Tang , J. Tang , G. Zeng , G. Yang , X. Xie , Y. Zhou , Y. Pang , Y. Fang , J. Wang , W. Xiong , Appl. Surf. Sci. 2015, 333, 220.

[79] A. Kaur , P. Chahal , T. Hogan , IEEE Electron Device Lett. 2016, 37, 142.

[80] G. Yu , L. Lyu , F. Zhang , D. Yan , W. Cao , C. Hu , RSC Adv. 2018, 8, 3312. 35541199 10.1039/c7ra12573a PMC9077499

[81] N. Koike , T. Ikuno , T. Okubo , A. Shimojima , Chem. Commun. 2013, 49, 4998. 10.1039/c3cc41904e 23609783

[82] Y. J. Wong , L. Zhu , W. S. Teo , Y. W. Tan , Y. Yang , C. Wang , H. Chen , J. Am. Chem. Soc. 2011, 133, 11422. 21732677 10.1021/ja203316q

[83] M. Baaden , M. Burgard , G. Wipff , J. Phys. Chem. B 2001, 105, 11131.

[84] J. Meng , C. He , Y. Li , J. Zhou , J. Li , C. Zheng , J. Zhao , T. Fujita , S. Ning , Y. Wei , Microporous Mesoporous Mater. 2021, 314, 110859.

[85] J. J. Meng , C. L. He , J. Zhou , T. Fujita , S. Y. Ning , Y. Z. Wei , J. Appl. Polym. Sci. 2021, 138, 49732.

[86] B. Yu , X. Jin , Y. Kuang , M. Megharaj , R. Naidu , Z. Chen , Chemosphere 2015, 141, 205. 26225434 10.1016/j.chemosphere.2015.07.050

[87] W. Zhang , H. Gao , J. He , P. Yang , D. Wang , T. Ma , H. Xia , X. Xu , Sep. Purif. Technol. 2017, 172, 158.

[88] L. Wang , J. Yang , Y. Li , J. Lv , J. Zou , Chem. Eng. J. 2016, 284, 1058.

[89] S. T. Le , A. Israpanich , T. Phenrat , Chemosphere 2022, 305, 135376. 35716714 10.1016/j.chemosphere.2022.135376

[90] Z. Luo , C. Wan , H. Xu , F. Zhao , Z. Jin , J. Mater. Sci.: Mater. Electron. 2020, 31, 5838.

[91] L. Sun , C. Han , N. Wu , B. Wang , Y. Wang , RSC Adv. 2018, 8, 13697. 35539358 10.1039/c8ra02164c PMC9079785

[92] C. S. R. Vusa , M. Venkatesan , A. K. , S. Berchmans , P. Arumugam , Sci. Rep. 2017, 7, 8354. 28827778 10.1038/s41598-017-08627-1 PMC5567138

[93] M. P. Rayaroth , T. P. Nguyen , Y. S. Chang , Materials Today: Proceedings 2020, 33, 1389.

[94] Y. Zhao , B. Yuan , Y. Shen , R. Hao , S. Yang , Environ. Sci. Pollut. Res. 2018, 25, 25526. 10.1007/s11356-018-2628-4 29959731

[95] A. T. Vu , T. N. Xuan , C. H. Lee , Journal of Water Process Engineering 2019, 28, 169.

[96] N. Farhadian , S. Liu , A. Asadi , M. Shahlaei , S. Moradi , J. Mol. Liq. 2021, 321, 114896.

[97] A. D. Becke , J. Chem. Phys. 1993, 98, 5648.

[98] K. Raghavachari , Theor. Chem. Acc. 2000, 103, 361.

[99] A. V. Marenich , C. J. Cramer , D. G. Truhlar , J. Phys. Chem. B 2009, 113, 6378. 19366259 10.1021/jp810292n

