# Supplementary material for: Serine Grafted Silica Coated Nanoscale Zero‐Valent Iron with Enhanced Fenton‐Like Degradation of Mixed Organic Solvents of Tributyl Phosphate and n‐dodecane
Source: Adv Sci (Weinh). 2025 Aug 11;12(41):e09319. doi: 10.1002/advs.202509319 (PMC12591147; doi:10.1002/advs.202509319)
Supplement: Supplementary file 1 — Supporting Information [file ADVS-12-e09319-s001.docx]

**Supporting Information**

**Serine grafted silica coated nanoscale zero-valent iron with enhanced Fenton-like degradation of mixed organic solvents of tributyl phosphate and n-dodecane**

Peijie Sun^a^, Haifeng Xu^a^, Lejin Xu ^a*^

^a^ Department of Nuclear Engineering and Technology, School of Energy and Power Engineering, Huazhong University of Science and Technology, Wuhan 430074, P R China

* Corresponding author

E-mail: [xulejin@hust.edu.cn](mailto:xulejin@hust.edu.cn) (L.J. Xu)

ORCID: 0000-0002-6488-5763

**Number of pages:** 13

**Number of figures:** 12

**Number of tables:** 2

**Number of text：**1


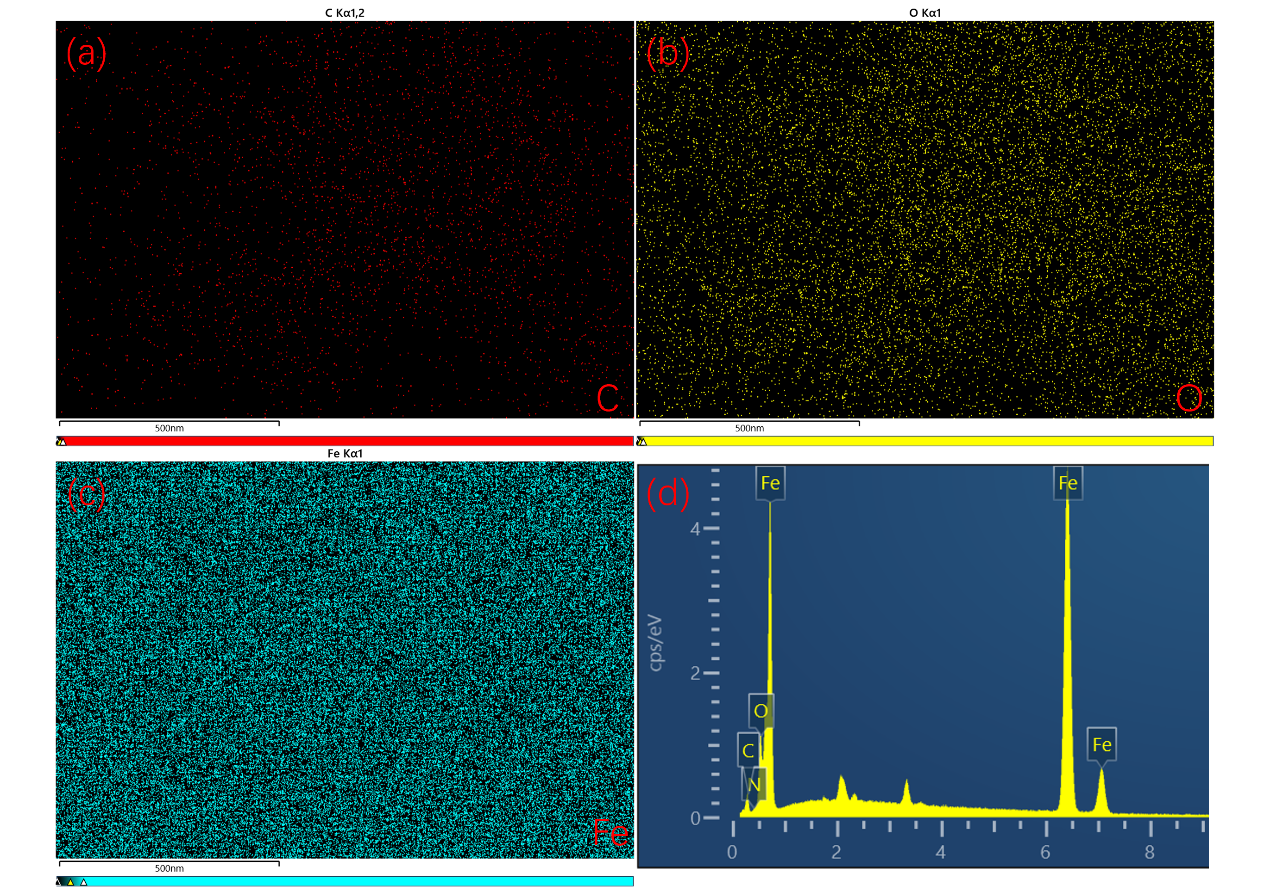


Fig. S1. SEM-EDS analysis and mapping graph of nZVI: (a-c) SEM-EDS elemental mapping of C, O and Fe; (d) EDS spectra.


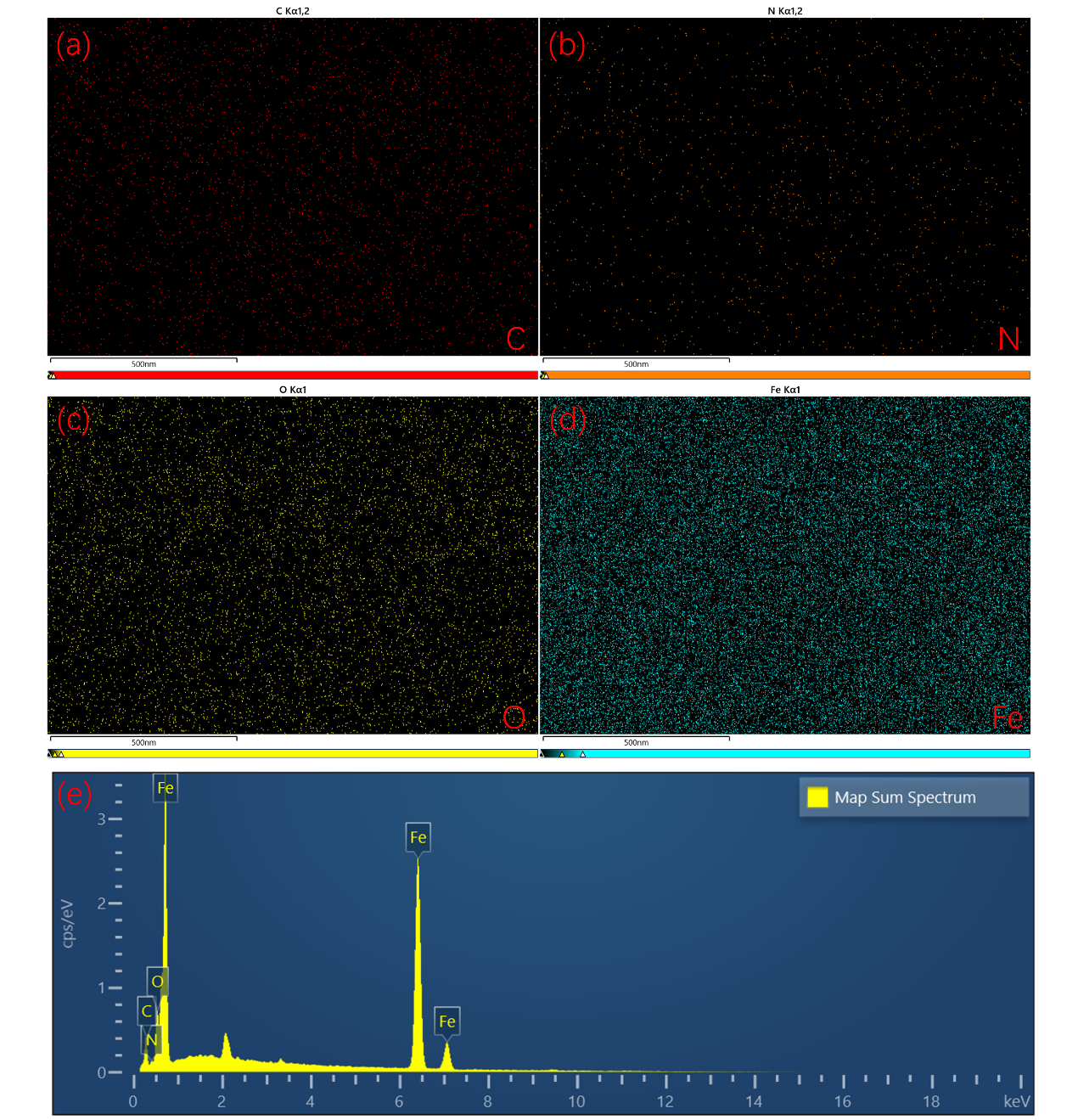


Fig. S2. SEM-EDS analysis and mapping graph of Ser-nZVI: (a-d) SEM-EDS elemental mapping of C, N, O and Fe; (e) EDS spectra.


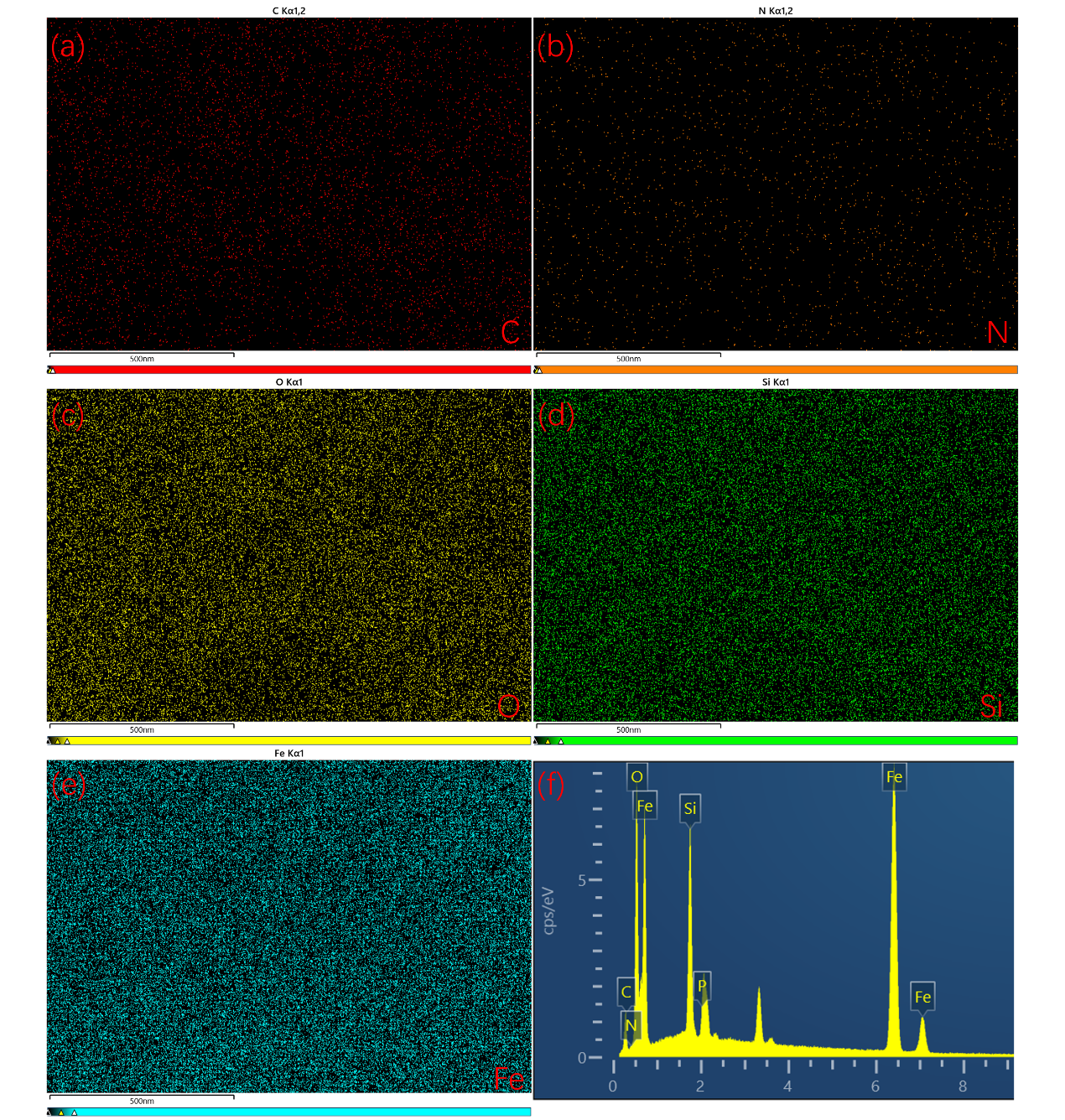


Fig. S3. SEM-EDS analysis and mapping graph of Ser-SiO_2_@nZVI: (a-e) SEM-EDS elemental mapping of C, N, O, Si and Fe; (f) EDS spectra.


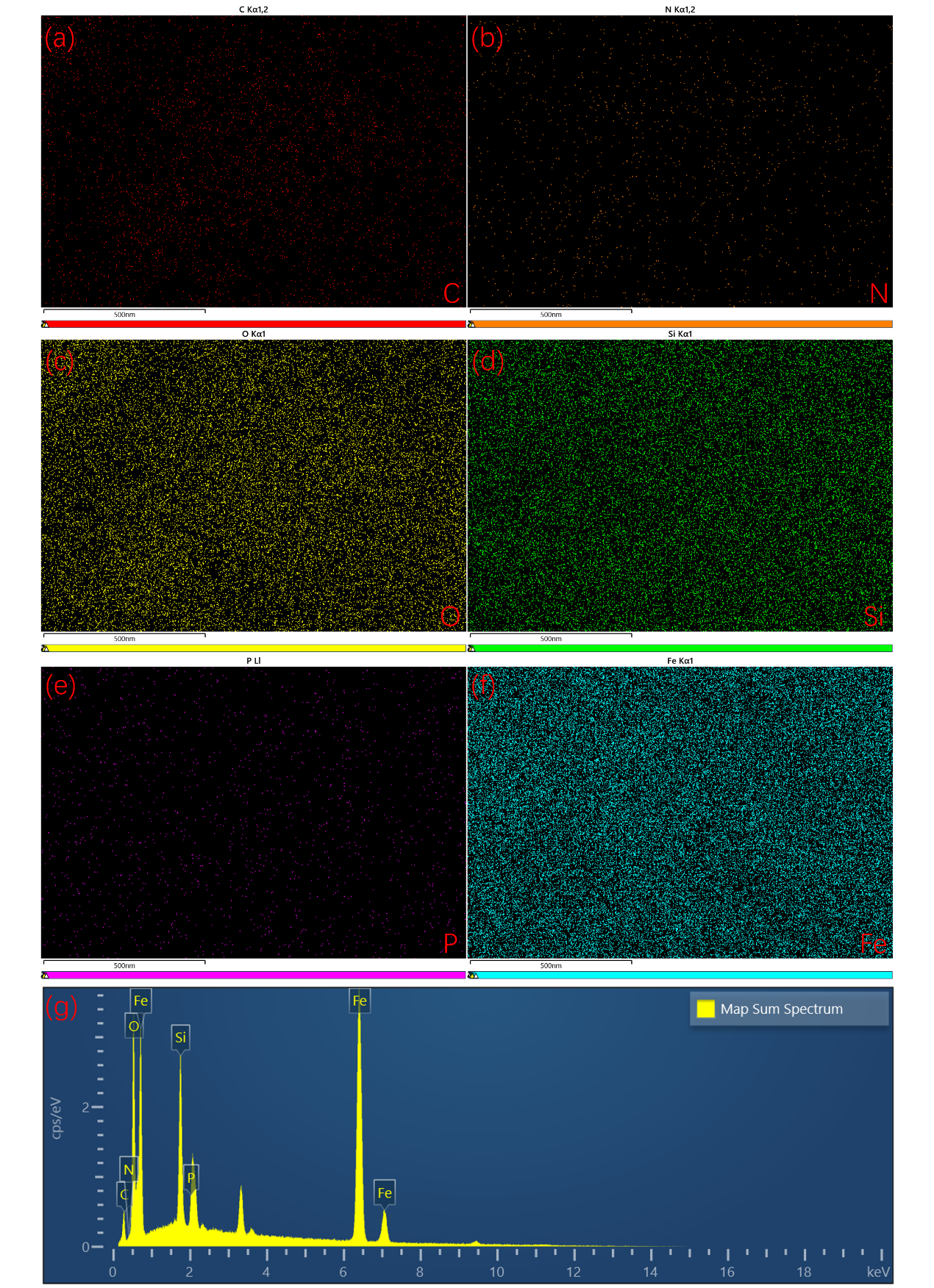


Fig. S4. SEM-EDS analysis and mapping graph of Ser-SiO_2_@nZVI after adsorption: (a-f) SEM-EDS elemental mapping of C, N, O, Si, P and Fe; (g) EDS spectra.


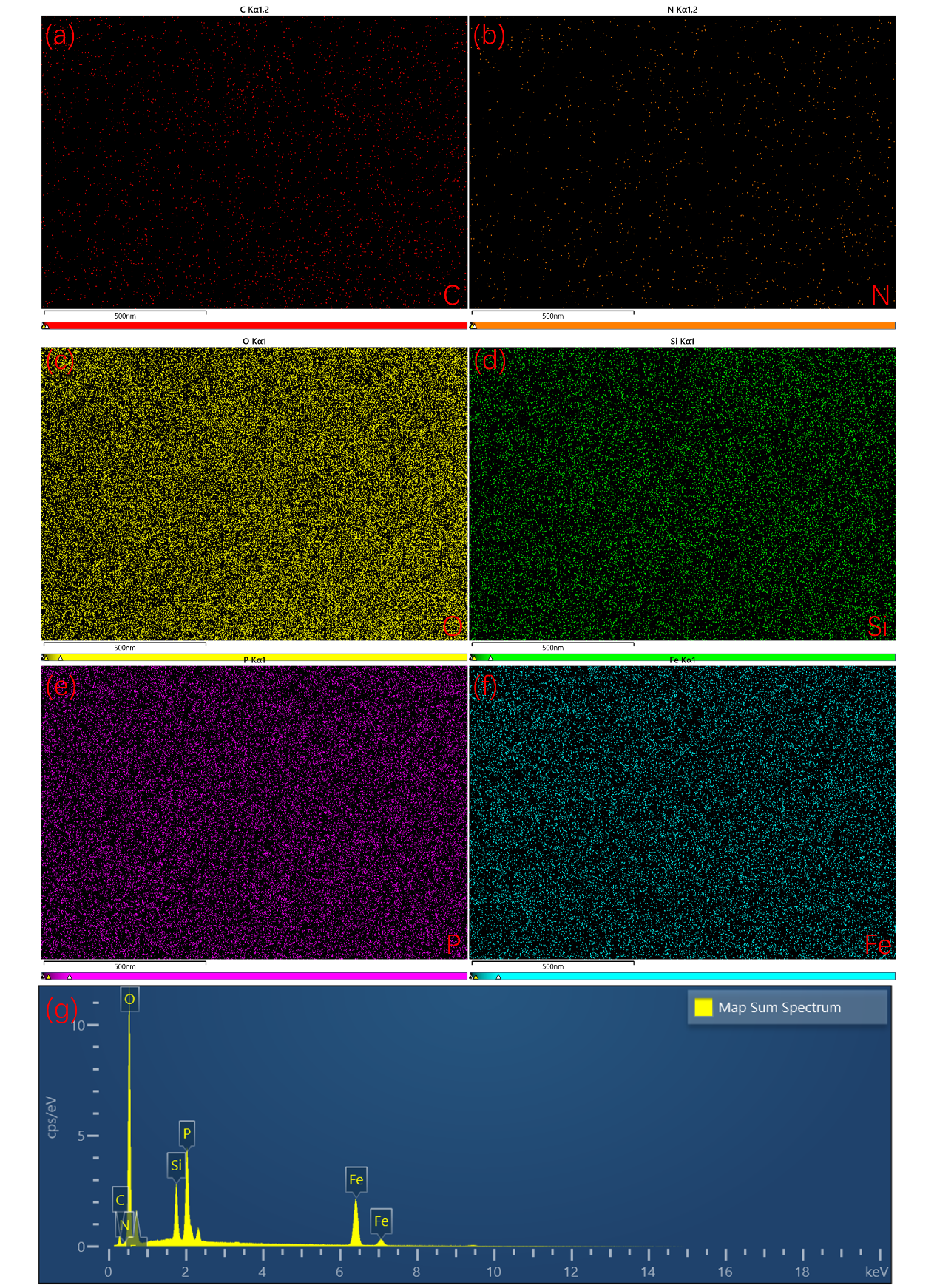


Fig. S5. SEM-EDS analysis and mapping graph of Ser-SiO_2_@nZVI after reaction: (a-f) SEM-EDS elemental mapping of C, N, O, Si, P and Fe; (g) EDS spectra.


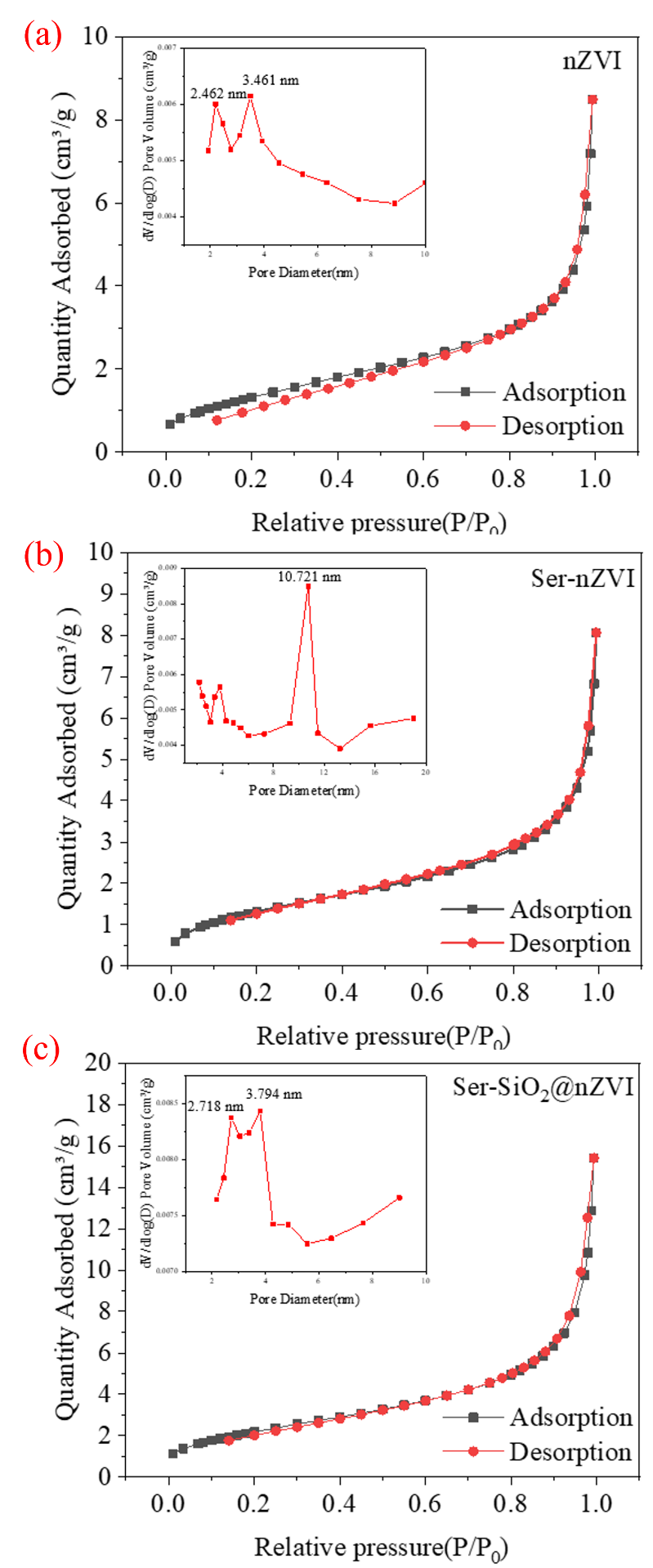


Fig. S6. Nitrogen adsorption-desorption isotherms and pore size distribution curves (inset) of (a) nZVI, (b) Ser-nZVI and (c) Ser-SiO_2_@nZVI.


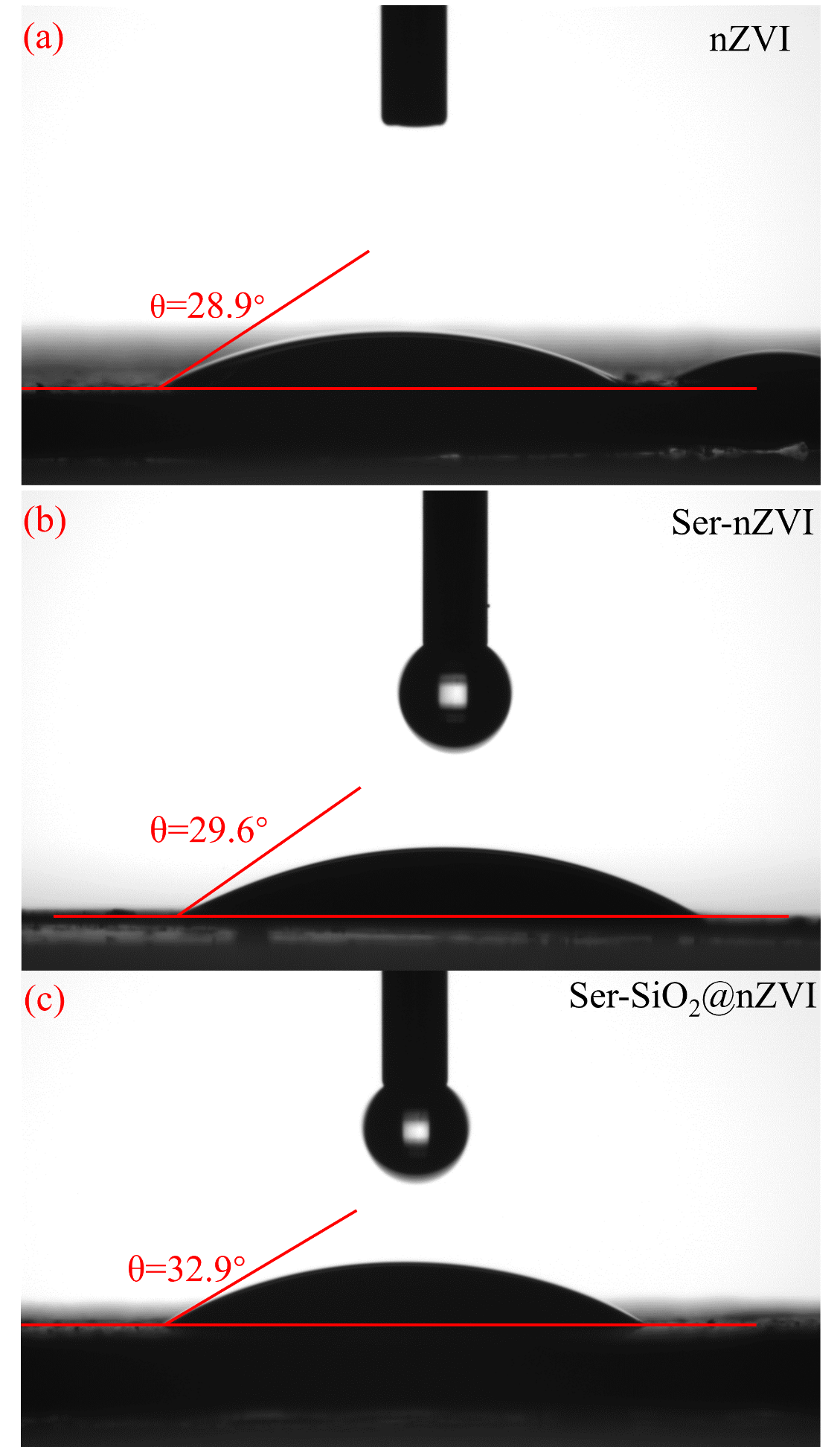


Fig. S7. Water contact angles of (a) nZVI, (b) Ser-nZVI and (c) Ser-SiO_2_@nZVI.

Fig. S8. FTIR spectra of Ser-SiO_2_@nZVI before, after adsorption and reaction


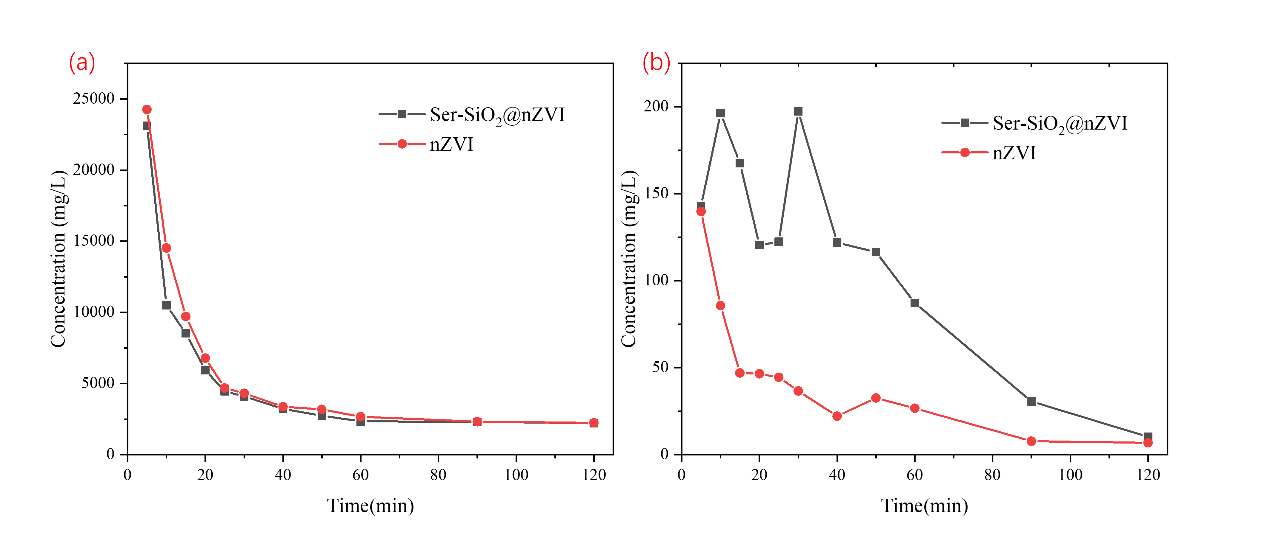


Fig. S9. The changes in the concentration of (a) Fe and (b) Fe²⁺ in the reaction solution at different catalysts.


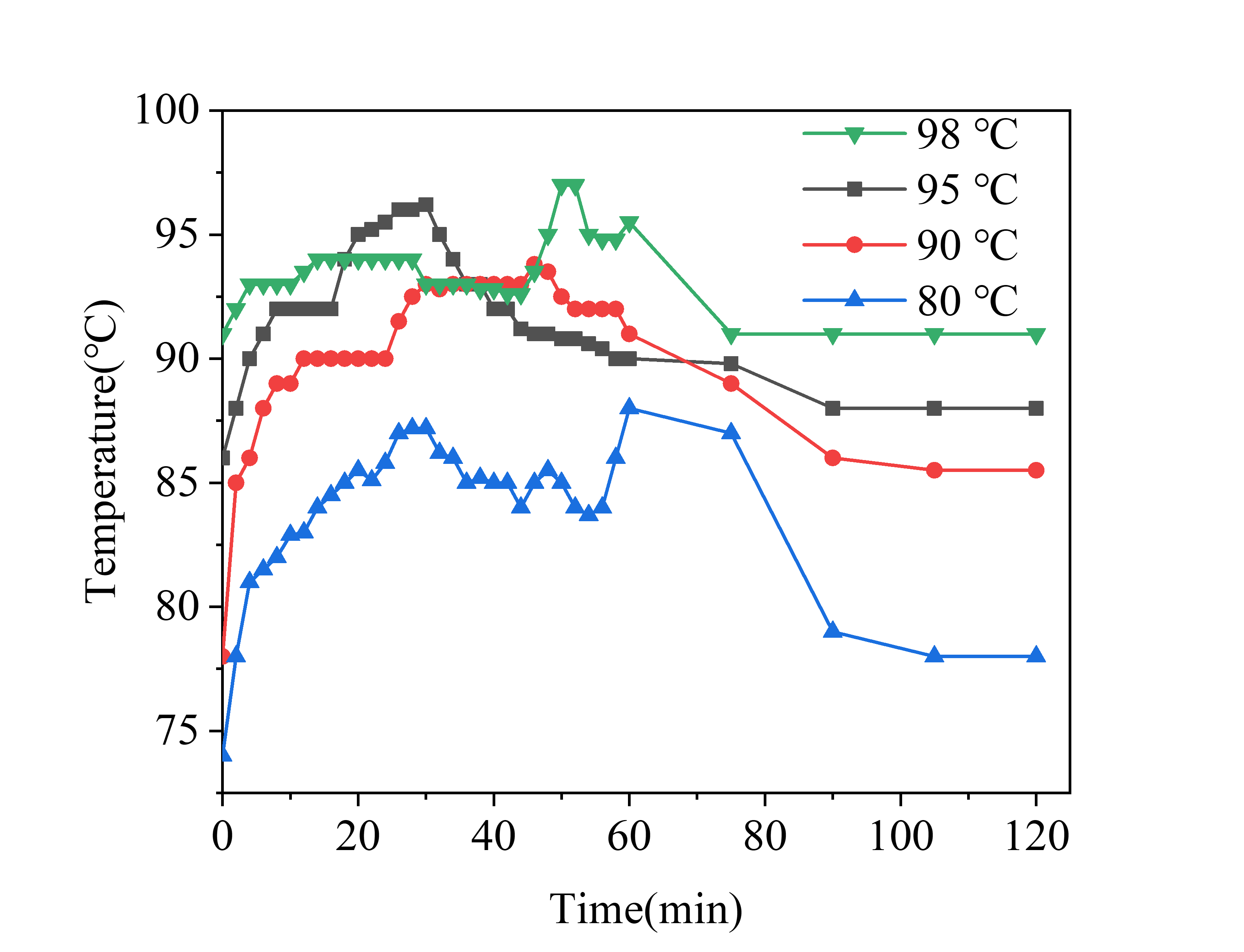


Fig. S10. Temperature changes during degradation at different temperatures.


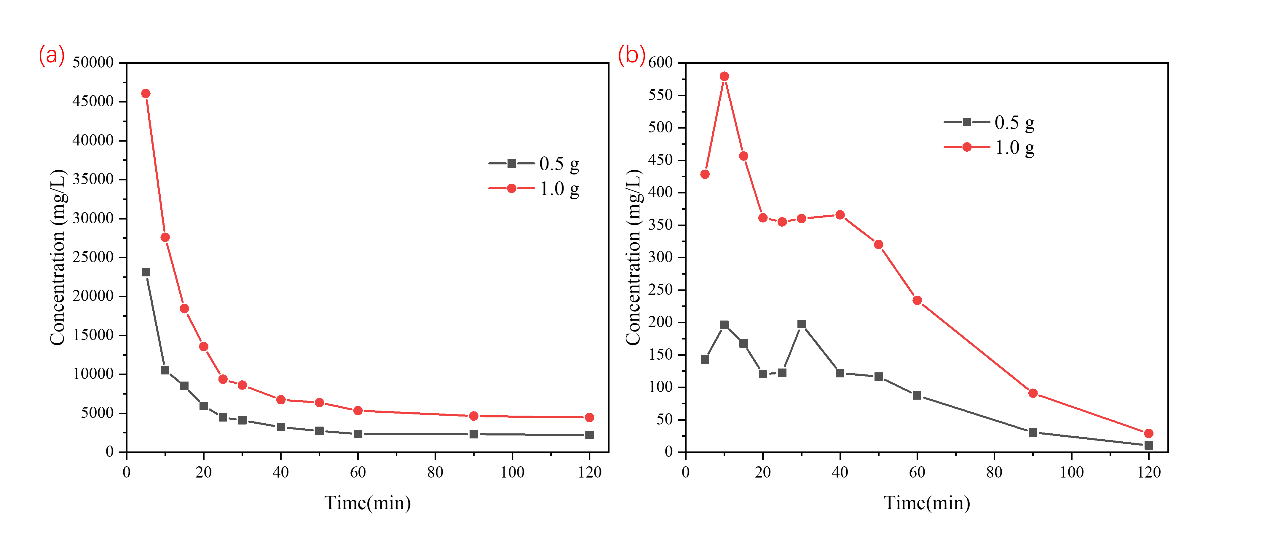


Fig. S11. The changes in the concentration of (a) Fe and (b) Fe²⁺ in the reaction solution at different dosages of Ser-SiO₂@nZVI


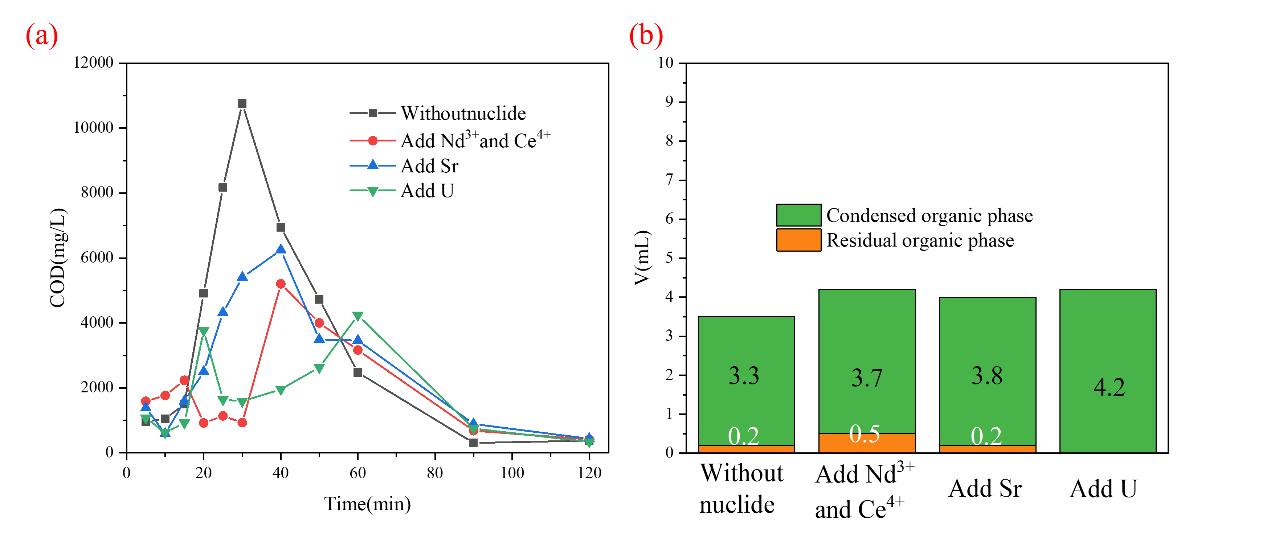


Fig. S12. Degradation of TBP and n-dodecane with and without nuclides: (a) COD of aqueous solution and (b) volume of organic phase.

Table S1. The specific surface areas and pore volumes of nZVI, Ser-nZVI and Ser-SiO_2_@nZVI

| Materials | nZVI | Ser-nZVI | Ser-SiO_2_@nZVI |
| --- | --- | --- | --- |
| Specific surface areas (m^2^/g) | 5.0452 | 4.9983 | 8.1944 |
| Pore volumes (cm^3^/g) | 0.014 | 0.013 | 0.024 |

Table S2. The energies of Ser-SiO_2_@nZVI+TBP system, Ser-SiO_2_@nZVI+*n*-dodecane system, Ser-nZVI+TBP system, Ser-nZVI+*n*-dodecane system, TBP, *n*-dodecane, Ser-SiO_2_@nZVI and Ser-nZVI structures, and the adsorption energies of TBP and *n*-dodecane on Ser-SiO_2_@nZVI and Ser-nZVI.

| *E* (TBP+Ser-SiO_2_@nZVI) | *E* (TBP) | *E* (Ser-SiO_2_@nZVI) | *E*_ads_ (kcal/mol) |
| --- | --- | --- | --- |
| -1211252.80 | -700284.39 | -510959.88 | -8.53 |
| *E* (TBP+Ser-nZVI) | *E* (TBP) | *E* (Ser-nZVI) | *E*_ads_ (kcal/mol) |
| -1028093.17 | -700284.39 | -327804.49 | -4.29 |
| *E* (*n*-dodecane+Ser-SiO_2_@nZVI) | *E* (*n*-dodecane) | *E* (Ser-SiO_2_@nZVI) | *E*_ads_ (kcal/mol) |
| -807801.59 | -296829.05 | -510959.88 | -12.66 |
| *E* (*n*-dodecane+Ser-nZVI) | *E* (*n*-dodecane) | *E* (Ser-nZVI) | *E*_ads_ (kcal/mol) |
| -624637.08 | -296829.05 | -327804.49 | -3.54 |

Text S1. Cost Estimation

According to the synthesis method in this study, to prepare 2.5 g of Ser-SiO_2_@nZVI, 11.12 g of FeSO_4_·7H_2_O, 10.8 g of KBH_4_, 0.01 mol of TEOS, 0.5 g of serine and other chemical reagents (such as ethanol) are required. Based on the prices of industrial-grade raw materials, ferrous sulfate heptahydrate costs 1600 yuan per ton, borohydride potassium costs 100,000 yuan per ton, TEOS costs 10,000 yuan per ton, and serine costs 150,000 yuan per ton. The total cost of the preparation materials is 1.79 yuan. Therefore, the estimated unit price for preparing Ser-SiO_2_@nZVI is 0.71 yuan/g.
